# Schistosomiasis–Microbiota Interactions: A Systematic Review and Meta-Analysis

**DOI:** 10.3390/pathogens13100906

**Published:** 2024-10-16

**Authors:** Philip Afful, Godwin Kwami Abotsi, Czarina Owusua Adu-Gyamfi, George Benyem, Gnatoulma Katawa, Samuel Kyei, Kathrin Arndts, Manuel Ritter, Kwame Kumi Asare

**Affiliations:** 1 Biomedical and Clinical Research Centre, College of Health and Allied Sciences, University of Cape Coast, Cape Coast, Ghana; afful.philip1997@gmail.com (P.A.); gabotsi@stu.ucc.edu.gh (G.K.A.); cagyamfi@stu.ucc.edu.gh (C.O.A.-G.); georgebenyem51@gmail.com (G.B.); skyei@ucc.edu.gh (S.K.); 2 Unité de Recherche en Immunologie et Immunomodulation (UR2IM)/Laboratoire de Microbiologie et de Contrôle de Qualité des Denrées Alimentaires (LAMICODA), Ecole Supérieure des Techniques Biologiques et Alimentaires, Université de Lomé, Lomé, Togo; mahkatawa@yahoo.fr; 3 Department of Optometry and Vision Science, College of Health and Allied Sciences, University of Cape Coast, Cape Coast, Ghana; 4 Institute for Medical Microbiology, Immunology, and Parasitology (IMMIP), University Hospital Bonn (UKB), 53127 Bonn, Germany; kathrin.arndts@ukbonn.de; 5 German-West African Centre for Global Health and Pandemic Prevention (G-WAC), Partner Site Bonn, 53127 Bonn, Germany; 6 Department of Biomedical Sciences, School of Allied Health Sciences, College of Health and Allied Sciences, University of Cape Coast, Cape Coast, Ghana; 7 Department of Immunology, Noguchi Memorial Institute for Medical Research, University of Ghana, Accra, Ghana

**Keywords:** schistosomiasis, microbiota, microbial diversity, immune modulation, dysbiosis

## Abstract

Introduction: Schistosomiasis, a tropical disease affecting humans and animals, affected 251.4 million people in 2021. *Schistosoma mansoni*, *S. haematobium*, *S. intercalatum*, and *S. japonicum* are primary human schistosomes, causing tissue damage, granulomas, ulceration, hemorrhage, and opportunistic pathogen entry. The gut and urinary tract microbiota significantly impact a host’s susceptibility to schistosomiasis, disrupting microbial balance; however, this relationship is not well understood. This systematic review and meta-analysis explores the intricate relationship between schistosomiasis and the host’s microbiota, providing crucial insights into disease pathogenesis and management. Methods: This systematic review used PRISMA guidelines to identify peer-reviewed articles on schistosomiasis and its interactions with the host microbiome, using multiple databases and Google Scholar, providing a robust dataset for analysis. The study utilized Meta-Mar v3.5.1; descriptive tests, random-effects models, and subgroups were analyzed for the interaction between Schistosomiasis and the microbiome. Forest plots, Cochran’s Q test, and Higgins’ inconsistency statistic (I^2^) were used to assess heterogeneity. Results: The human *Schistosoma* species were observed to be associated with various bacterial species isolated from blood, stool, urine, sputum, skin, and vaginal or cervical samples. A meta-analysis of the interaction between schistosomiasis and the host microbiome, based on 31 studies, showed 29,784 observations and 5871 events. The pooled estimates indicated a significant association between schistosomiasis and changes in the microbiome of infected individuals. There was considerable heterogeneity with variance effect sizes (*p* < 0.0001). Subgroup analysis of *Schistosoma* species demonstrated that *S. haematobium* was the most significant contributor to the overall heterogeneity, accounting for 62.1% (*p* < 0.01). *S. mansoni* contributed 13.0% (*p* = 0.02), and the coinfection of *S. haematobium* and *S. mansoni* accounted for 16.8% of the heterogeneity (*p* < 0.01), contributing to the variability seen in the pooled analysis. Similarly, praziquantel treatment (RR = 1.68, 95% CI: 1.07–2.64) showed high heterogeneity (Chi^2^ = 71.42, df = 11, *p* < 0.01) and also indicated that *Schistosoma* infections in males (RR = 1.46, 95% CI: 0.00 to 551.30) and females (RR = 2.09, 95% CI: 0.24 to 18.31) have a higher risk of altering the host microbiome. Conclusions: Schistosomiasis significantly disrupts the host microbiota across various bodily sites, leading to increased susceptibility to different bacterial taxa such as *E. coli*, *Klebsiella*, *Proteus*, *Pseudomonas*, *Salmonella*, *Staphylococcus*, *Streptococcus*, and *Mycobacterium* species (*M. tuberculosis* and *M. leprae*). This disruption enables these bacteria to produce toxic metabolites, which in turn cause inflammation and facilitate the progression of disease. The impact of schistosomiasis on the vaginal microbiome underscores the necessity for gender-specific approaches to treatment and prevention. Effective management of female genital schistosomiasis (FGS) requires addressing both the parasitic infection and the resulting microbiome imbalances. Additionally, praziquantel-treated individuals have different microbiome compositions compared to individuals with no praziquantel treatment. This suggests that combining praziquantel treatment with probiotics could potentially decrease the disease severity caused by an altered microbiome.

## 1. Introduction

Schistosomiasis, a tropical disease affecting both humans and animals, necessitated preventive treatment for 251.4 million people in 2021, as reported by the World Health Organization (WHO) [1]. The disease, primarily endemic in tropical and subtropical regions, poses significant public health challenges and requires efforts to manage and reduce its impact. *Schistosoma mansoni* Sambon, 1907, *Schistosoma haematobium* Bilharz, 1852, *Schistosoma intercalatum* Fisher, 1934, and *Schistosoma japonicum* Katsurada, 1904 are the primary schistosomes infecting humans, leading to intestinal, hepatic, and urinary schistosomiasis across regions such as Africa, the Arabian Peninsula, South America, China, the Philippines, and Indonesia [2]. Schistosomes, parasitic worms, infect humans through contact with contaminated water, causing host damage at various life cycle stages [3,4]. Their secretions and excretions elicit immune responses that can lead to a range of complications [5]. Adult worms of *S. japonicum*, *S. intercalatum*, and *S. mansoni* typically inhabit the portal-mesenteric venous system, where they lay eggs, contributing to intestinal and hepatic schistosomiasis [3,6]. In contrast, *S. haematobium* primarily resides in the bladder plexus to lay its eggs, causing urinary schistosomiasis [7].

Schistosome infections cause significant tissue damage, particularly through the deposition of eggs in the intestinal and urinary walls [8,9]. The eggs secrete antigenic mixtures that attract immune cells and trigger the infiltration of inflammatory factors [9]. This immune response leads to the formation of granulomas, which are clusters of immune cells that form around the eggs [10,11]. The granulomas can cause tissue damage, leading to ulceration and hemorrhage [11]. These lesions disrupt the normal tissue barriers and alter the local immune environment, which can facilitate the entry and growth of opportunistic pathogens, thereby influencing the diversity of microbial infections [12,13]. The compromised tissue integrity and the immunomodulatory effects of schistosomiasis create a conducive environment for the proliferation of various bacterial species, potentially leading to coinfections and complicating the clinical management of schistosomiasis [8,14].

Female genital schistosomiasis (FGS) is caused by the entrapment of *Schistosoma haematobium* eggs in genital tissues, which is associated with a range of adverse reproductive outcomes, including ectopic pregnancy, infertility, low birth weight, preterm delivery, and various other reproductive health abnormalities [15,16]. FGS is known to play a significant role in sexually transmitted infections (STIs). It causes dysbiosis in the optimal lactic acid-producing lactobacilli microbiota, leading to the harboring of nonoptimal microbiota, such as those associated with bacterial vaginosis (BV) and vulvovaginal candidiasis [17]. High-intensity urinary *S. haematobium* infections can alter the diversity of the cervicovaginal microbiota, which is crucial for maintaining reproductive health. FGS-associated BV infections can induce pelvic inflammatory disease, which has been linked to an increased risk of STIs, including syphilis, gonorrhea, chlamydia, and trichomoniasis, as well as incurable viral infections such as herpes simplex virus (HSV), human immunodeficiency virus (HIV), and human papillomavirus (HPV) [18]. The presence of *S. haematobium* eggs and the resulting inflammation may disrupt the mucosal barrier and immune response, facilitating the invasion and persistence of pathogenic microorganisms. This disruption compromises the natural defense mechanisms of the genital tract, making it more susceptible to infections and causes urinary tract infections (UTIs) and other reproductive health issues. Thus, understanding FGS and its impact on the vaginal microbiome could improve diagnosis, treatment, and prevention strategies, as well as broader public health initiatives to reduce the prevalence of schistosomiasis and associated reproductive health complications.

The mammalian gut harbors a diverse community of microbiota that play crucial roles in regulating host immunity and physiology [19]. These microorganisms are essential for maintaining homeostasis and contribute significantly to the host’s defense mechanisms against pathogens [20,21]. They help modulate the immune system, ensuring a balanced response to infections and preventing excessive inflammation that could damage tissues [22]. In the context of schistosome infections, the gut microbiota influence the host’s susceptibility by regulating immune responses and maintaining the integrity of the gut barrier [23]. A healthy microbiome supports a robust immune system capable of mounting effective defenses against schistosome larvae, potentially limiting their establishment and migration within the host [24,25]. Thus, the composition and diversity of both the gut and urinary tract microbiota are critical in shaping the host’s susceptibility to schistosomiasis, highlighting the intricate relationship between gut or urinary tract health and parasitic infections [26,27].

Praziquantel, the primary treatment for schistosomiasis, is effective but can cause several side effects and impact the gut microbiome [28]. Common side effects include dizziness, headache, nausea, abdominal pain, and fatigue, while less common effects include allergic reactions, fever, vomiting, diarrhea, and muscle pain [29]. Thus, praziquantel treatment can disrupt the microbial diversity of the gut microbiome, reducing the populations of beneficial bacteria like *Lactobacillus acidophilus* Moro, 1900 *or Lactobacillus casei* Orla-Jensen, 1916 *and Bifidobacterium longum* Reuter, 1963 *or Bifidobacterium bifidum* Tissier, 1900 and potentially allowing pathogenic bacteria to thrive [27]. This disruption can alter the host’s immune response and metabolic processes, affecting nutrient absorption and overall health. Long-term alterations in gut flora due to praziquantel treatment can increase the risk of gastrointestinal conditions such as irritable bowel syndrome (IBS) and inflammatory bowel disease (IBD) [30].

Thus, the interplay between schistosome infections and the host’s microbial flora is an intricate aspect of the disease’s pathogenesis [27]. Schistosome parasitic flatworm and its treatment can induce changes in the host’s microbiota, potentially altering the balance of microbial communities within the body [26,27]. Microbial diversity is crucial for maintaining various physiological functions, including metabolic processes and immune regulation [31]. When the diversity of microbial flora is high, it often indicates a healthier state, as different species contribute to different functions, such as nutrient metabolism and immune modulation [32]. This diverse microbial community helps to maintain homeostasis and resist the colonization of pathogenic organisms [33]. However, schistosome infections can disrupt this delicate balance [5]. The presence of the parasite in the host’s body can trigger immune responses and inflammatory reactions, which may inadvertently affect the composition and function of the microbiota [34]. This disruption can lead to dysbiosis, where there is an imbalance in the microbial community, with potentially harmful consequences [35,36].

Dysbiosis resulting from schistosome infections can have several detrimental effects on the host [26]. It may compromise organ integrity, impair metabolic processes, and weaken immune competence [37]. These effects can exacerbate the impact of schistosomiasis, leading to more severe health outcomes and complications [38]. Thus, understanding the relationship between schistosome infections and the host’s microbial flora is essential for developing effective strategies for managing and treating schistosomiasis by targeting the microbial imbalance induced by the parasite and may lead to the identification of novel approaches to modulate the host’s immune response and mitigate the severity of the disease. Additionally, interventions aimed at restoring microbial diversity could potentially enhance the effectiveness of conventional treatments for schistosomiasis. This systematic review and meta-analysis aims to examine the interactions between schistosomiasis and the host’s microbiota and will provide valuable insights into the complex relationship between these factors and their implications for disease pathogenesis and management.

## 2. Materials and Methods

### 2.1. Literature Search Strategy

The literature search strategy for this systematic review adhered to the Preferred Reporting Items for Systematic Reviews and Meta-Analyses (PRISMA) guidelines with the PRISMA checklist (Table S1) [39] to ensure a comprehensive identification of peer-reviewed articles examining schistosomiasis and its interactions with the host microbiome. The protocol for this review was registered in the International Prospective Register of Systematic Reviews (PROSPERO), Centre for Reviews and Dissemination (CRD) at the University of York (Registration Number CRD42024589163) on the 12th September 2024. The search encompassed multiple electronic databases, including Scopus, PubMed, Medline, Science Direct, and Cochrane, covering publications from January 1960 to May 2024. The search terms were crafted using the Boolean operator “AND” to combine relevant keywords such as “schistosomiasis AND microbiome”, “schistosomiasis AND bacteria”, “*Schistosoma* AND gut microbiota”, and “schistosomiasis AND host interaction.” Only articles available in full text, published in English, and openly accessible were included to ensure comprehensive data availability (Table S2). Additionally, a manual search was conducted via Google Scholar to identify any pertinent studies not indexed in the primary databases. The selection process involved an initial screening of titles and abstracts to ensure relevance, followed by a detailed assessment of the full texts of qualifying articles. Furthermore, the reference lists of selected studies were examined to identify additional relevant citations. This rigorous and systematic approach facilitated the compilation of a robust dataset on schistosomiasis and its interactions with the host microbiome, enabling a thorough analysis of the relationship between schistosomiasis and the host microbiota.

### 2.2. Study Eligibility Criteria

The study employed a rigorous screening and selection process to identify relevant articles on the interaction between schistosomiasis and the microbiome. The primary focus was on original research articles that included human participants from various demographics and geographic regions. This approach was designed to gather comprehensive and diverse data. The inclusion criteria mandated that studies must be original research articles involving human subjects of any age, gender, race, or location, specifically examining the interaction between schistosomiasis and the microbiome. To maintain the integrity of the analysis, only articles published in English were considered, ensuring that the language barrier did not impede the accurate interpretation of the data. This included experimental studies on non-human subjects, review articles, letters to editors, and duplicate studies, which do not contribute original data or analysis. Additionally, any articles lacking the relevant keywords or deemed irrelevant to the study’s aims were excluded from consideration. This methodical approach ensured that only high-quality, pertinent articles were selected, thereby contributing to a meaningful and comprehensive understanding of the interaction between schistosomiasis and the microbiome.

### 2.3. Study Selection and Data Extraction

The study employed a systematic and thorough methodology for selecting articles and extracting data to investigate the interaction between schistosomiasis and the microbiome. The process began with an exhaustive search across multiple databases to identify pertinent articles, followed by the removal of duplicates to ensure a clean dataset. This initial step aimed to streamline the review process and focus on unique and relevant studies. Subsequently, two of the authors served as independent reviewers and meticulously screened the articles to assess their adherence to predefined inclusion criteria. This dual-review approach minimized potential biases and ensured the rigorous selection of articles. During the full-text review phase, the reviewers extracted essential data points from each selected article, including publication details, study settings, population characteristics, and key results about the interaction between schistosomiasis and the microbiome. By systematically extracting relevant information, the study aimed to capture a comprehensive overview of the topic. The involvement of two independent reviewers in the screening and data extraction processes bolstered the study’s credibility and minimized the risk of errors or subjective interpretations. Any discrepancies between the reviewers were resolved through discussion or consultation with a third reviewer, further enhancing the study’s robustness. Overall, this systematic and structured approach ensured the thorough collection and analysis of data, facilitating a reliable examination of the interaction between schistosomiasis and the microbiome.

### 2.4. Assessment of Study Quality and Risk of Bias

The study employed the Joanna Briggs Institute (JBI) critical appraisal checklist guidelines (Table S3) to assess the quality of the selected articles, reflecting a commitment to rigorously evaluating methodological soundness and risk of bias [40]. This checklist (Table S3) offers a structured framework for evaluating various aspects of study quality, encompassing methodology, sample representativeness, data analysis, and result interpretation. The involvement of two independent reviewers in conducting the quality assessment aligns with best practices, aiming to reduce bias and enhance the reliability of the evaluation process. This approach ensures that diverse perspectives are considered, thereby mitigating the influence of subjective judgment on the assessment outcome. Moreover, any discrepancies or disagreements between the reviewers were resolved through consensus discussions, underscoring the study’s dedication to maintaining consistency and rigor in the evaluation process. By adhering to the JBI critical appraisal checklist guidelines (Table S3) and incorporating the input of two independent reviewers, the study ensured a standardized and systematic approach to evaluating study quality and the risk of bias. The process involves applying nine criteria, with each rated as “YES” or “NO”. A scoring system was then used to assign a numerical value to each study based on the number of criteria met. Studies were categorized as low quality (scores 0–4), moderate quality (scores 5–7), or high quality (scores 8–9) (Table S4). This rigorous methodological framework not only enhances the validity and reliability of the study findings but also instills confidence in the robustness of the included literature and the conclusions drawn from the analysis.

### 2.5. Data Analysis

The study implemented a meticulous and comprehensive statistical approach to analyze data collected from various sources regarding the interaction between schistosomiasis and the microbiome. Data management and organization were conducted using Microsoft Excel, ensuring a structured and systematic process. For statistical analysis, Meta-Mar v3.5.1, a specialized tool tailored for meta-analysis calculations, was utilized (https://meta-mar.shinyapps.io/meta-analysis-calculator/) (accessed on 17 March 2024) [41]. This software provides a range of statistical tests and procedures specifically designed for meta-analytical studies. Descriptive statistical tests, including dichotomous models for risks and ratios, as well as average effect size using log risk ratio and log risk difference, were applied to enable a comprehensive assessment of the data. A random-effects model was employed to calculate pooled datasets, considering the anticipated variability between studies. This model provides a more conservative estimate of the overall effect size while acknowledging potential heterogeneity across studies. Subgroup analyses were conducted based on *Schistosoma* species, praziquantel treatment, and gender, allowing for the exploration of variations in the interaction between schistosomiasis and the microbiome. This approach enhances the granularity of the analysis and provides insights into potential geographical, treatment-, and gender-specific differences in disease epidemiology. To assess heterogeneity among studies, various statistical methods were utilized, including visual inspection of forest plots, Cochran’s Q test, and Higgins’ inconsistency statistic (I^2^). An I^2^ value above 50% was considered indicative of substantial heterogeneity, prompting further exploration of potential sources of variation between studies. Overall, this rigorous statistical approach ensured a robust analysis of the interaction between schistosomiasis and the microbiome, providing valuable insights into the epidemiology and interaction of these infections.

### 2.6. Publication Bias

The study adopted proactive measures to address publication bias and heterogeneity, crucial steps in ensuring the robustness and reliability of its findings. Publication bias, the tendency for studies with significant results to be published more readily than those with non-significant results, was rigorously evaluated through several methods. Firstly, the study utilized the Fail-safe N calculation using the Rosenthal Approach. This method estimates the number of unpublished or missing studies required to nullify the observed effect, providing insight into the potential impact of publication bias on the results. Additionally, funnel plots were employed to visually assess the symmetry of the distribution of effect sizes. Asymmetry in funnel plots can indicate publication bias, with Egger’s test providing a statistical evaluation of this asymmetry [42]. Furthermore, Higgins’ I^2^ statistic was utilized to assess the degree of heterogeneity between studies. This statistic quantifies the proportion of total variation across studies due to heterogeneity rather than chance, with higher values indicating greater heterogeneity. Sensitivity analyses were also conducted to examine the influence of the largest studies on the meta-analyses, allowing for an exploration of potential sources of variation and bias. Overall, these comprehensive approaches provided a thorough evaluation of potential biases and variations in the data, strengthening the validity and credibility of the study’s conclusions regarding the interaction between schistosomiasis and the microbiome.

## 3. Results

### 3.1. Study Characteristics

**Figure 1:**
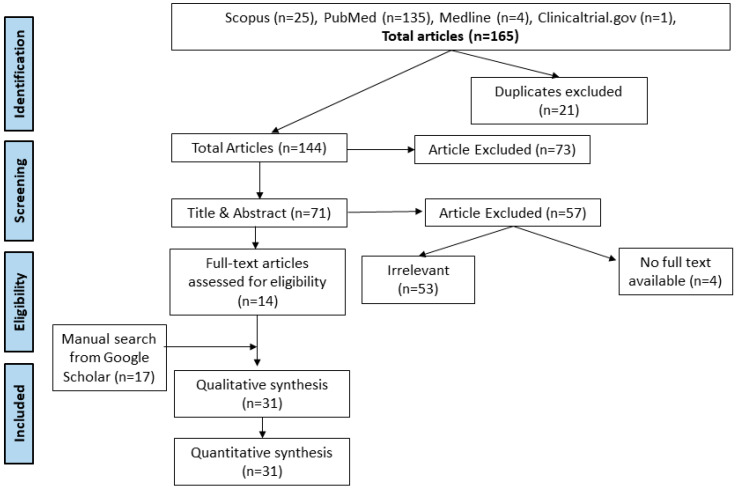
PRISMA flow chart for search and selection of included studies.

**Figure 2:**
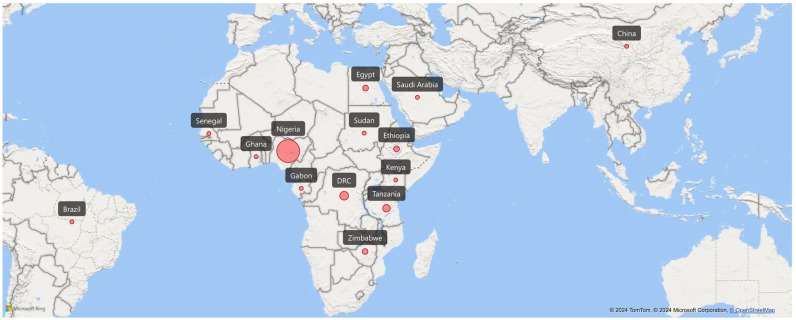
The geographical distribution of the included studies. The red indicates the various studies and the countries where they were conducted.

A comprehensive search of six electronic databases, including Scopus, PubMed, Medline, and ClinicalTrials.gov, resulted in the identification of 165 articles. After removing duplicates and screening titles and abstracts, 14 full-text articles were deemed eligible for inclusion. Additionally, a manual search on Google Scholar yielded 17 more articles, resulting in a total of 31 studies included in the quantitative synthesis (Figure 1) [43,44,45,46,47,48,49,50,51,52,53,54,55,56,57,58,59,60,61,62,63,64,65,66,67,68,69,70,71,72,73]. The *Schistosoma* species (*S. haematobium*, *S. japonicum*, *S. mansoni*, *S. intercalatum*, and *S. haematobium—S. mansoni* coinfection) were observed to be associated with various bacterial species, including *Escherichia coli*, *Staphylococcus aureus*, *Klebsiella* spp., *Yersinia enterocolitica*, *Enterobacter aerogenes*, *Salmonella enterica*, *Pseudomonas aeruginosa*, *Salmonella typhi*, *Helicobacter pylori*, and *Citrobacter* spp., among others. These bacterial species were isolated from blood, stool, urine, sputum, skin, and vaginal or cervical samples. Interestingly, *Escherichia* was isolated from 12 of the included studies [43,44,45,47,48,49,50,51,55,56,70,71], *Klebsiella* from 11 studies [43,44,45,47,48,49,50,51,55,70,71], *Staphylococcus* [43,47,48,49,51,56,70,71] and *Salmonella* [48,51,53,54,56,57,63,67] both from eight studies each, *Pseudomonas* from six studies [43,44,48,49,55,70], *Proteus* from five studies [43,48,49,50,70], and *Streptococcus* [44,49,56,70] and *Mycobacterium* [59,65,66,68] from four studies each. The rest of the bacterial isolates were reported in one or two studies. Most of the studies originated in Africa, where schistosomiasis remains endemic, with fewer studies conducted in Brazil, China, and Saudi Arabia. (Table 1, Figure 2). The geographical distribution of the included studies is shown in Figure 2, highlighting a significant concentration of research in African countries, reflecting the endemic nature of schistosomiasis in the region.

### 3.2. Schistosomiasis Interactions with the Host Microbiome

**Figure 3:**
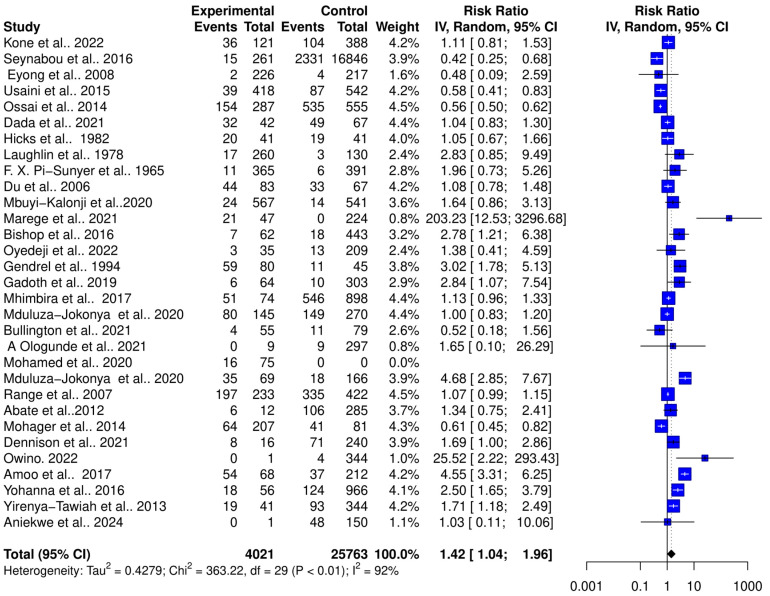
Forest plot showing the risk ratio of schistosomiasis and bacterial infections from 31 studies.

**Figure 4:**
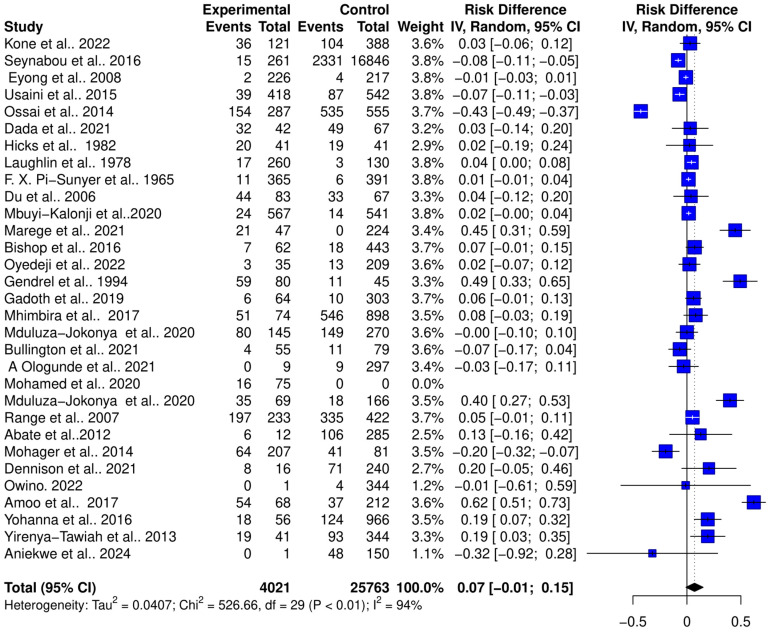
Forest plot showing the risk difference of schistosomiasis and bacterial infections from 31 studies.

The interaction between schistosomiasis and the host microbiome was analyzed through a meta-analysis of 31 studies, comprising 29,784 observations and 5871 events. The pooled estimates indicated a significant association between schistosomiasis and changes in the microbiome of infected individuals. The relative risk (RR) for this association was found to be 1.42, with a 95% confidence interval (CI) of 1.04 to 1.96, yielding a statistically significant result (z/t = 2.27, *p* = 0.0307) under the random-effects model. This result suggests that individuals with schistosomiasis are 42% more likely to experience significant changes in their microbiome compared to those without the infection (Figure 3). The analysis revealed considerable heterogeneity in the interactions between schistosomiasis and the host microbiome, as evidenced by several statistical measures: τ^2^: 0.4279 (with a range from 0.2907 to 1.6277), which is an indicator of variance among the effect sizes; τ: 0.6541 (ranging from 0.5391 to 1.2758), representing the standard deviation of the true effect sizes; I^2^: 92.0% (with a range from 89.7% to 93.8%), indicating a high percentage of variability due to between-study differences rather than chance; H: 3.54 (ranging from 3.11 to 4.02), showing the ratio of total variability to within-study variability; and Cochran’s Q: 363.22, with a *p*-value of less than 0.0001, highlighting significant heterogeneity among the studies (Figure 3). This high level of heterogeneity indicates that the studies varied widely in their findings, suggesting that factors such as geographic location, population characteristics, *Schistosoma* species, praziquantel treatment, and gender or study methodologies could have influenced the outcomes. Additionally, the risk difference (RD) analysis across the pooled estimates did not show a significant change in the risk or proportion of interactions between schistosomiasis and the microbiome across the studies. The RD for the random-effects model was 0.0704 (95% CI, −0.0092 to 0.15), and the z/t value was 1.81 with a *p*-value of 0.0807, indicating that there was no statistically significant risk difference in the altered microbiome across the studies (Figure 4). The heterogeneity in the risk difference analysis was also significant, with τ^2^: 0.0407 (ranging from 0.0234 to 0.0756), τ: 0.2019 (ranging from 0.1531 to 0.2750), I^2^: 94.5% (ranging from 93.1% to 95.6%), H: 4.26 (ranging from 3.80 to 4.78), and Cochran’s Q: 526.66, with a *p*-value of less than 0.0001, further emphasizing the significant heterogeneity (Figure 4). The substantial heterogeneity (I^2^ = 94.5%) observed in the risk difference suggests that a large portion of the variability in the interaction between schistosomiasis and the microbiome cannot be solely attributed to sampling error but rather to true differences across the studies. The high levels of heterogeneity identified in both the relative risk and risk difference analyses imply that schistosomiasis and its impact on the microbiome are influenced by a variety of factors. These factors might include differences in environmental conditions, and local microbiome compositions could significantly affect how schistosomiasis interacts with the host microbiome; genetic diversity and differences in health status among populations could lead to varied responses to schistosomiasis; and differences in study design, data collection, and microbiome analysis techniques could contribute to the observed heterogeneity. Overall, the analysis highlights the complex and variable nature of schistosomiasis interactions with the host microbiome.

### 3.3. Schistosoma Species and Their Interactions with the Host Microbiome

**Figure 5:**
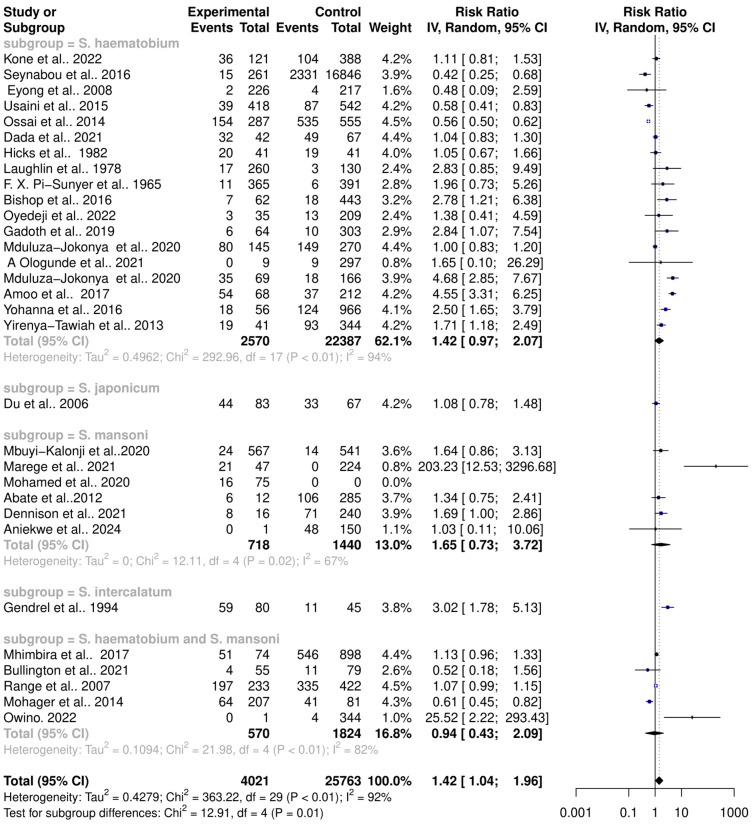
Forest plot showing the risk ratio of schistosomiasis and bacterial infection based on *Schistosoma* species.

**Figure 6:**
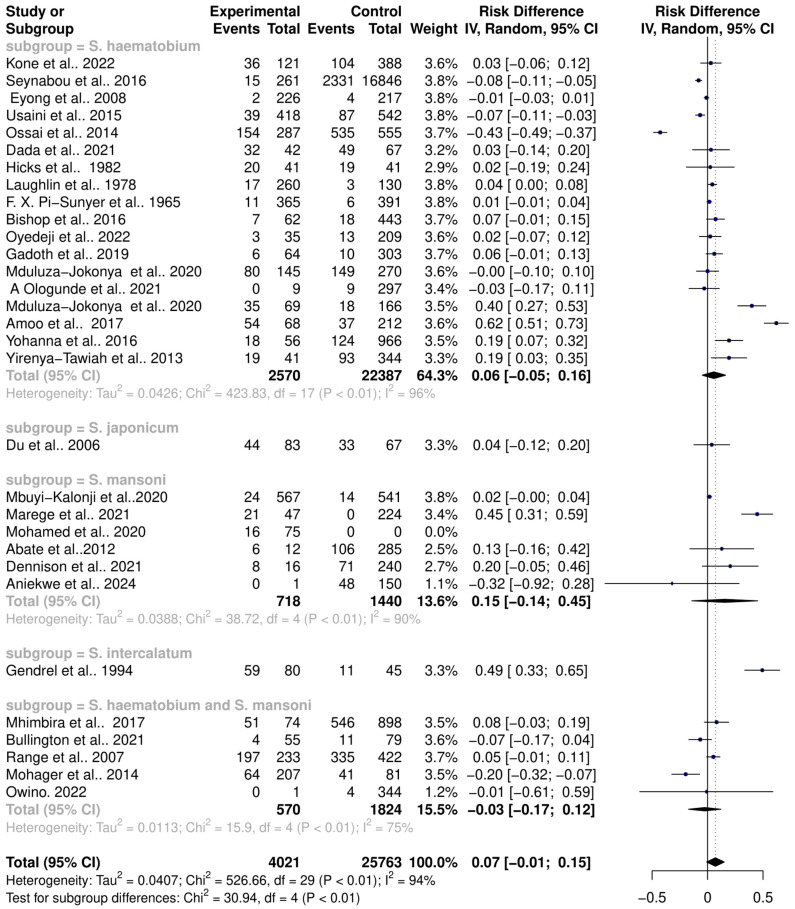
Forest plot showing the risk difference of schistosomiasis and bacterial infection based on *Schistosoma* species.

The pooled estimates of interactions between various *Schistosoma* species and the host microbiome indicate substantial variance across different study groups. This variance suggests that each *Schistosoma* species may uniquely interact with the host microbiome, contributing to the significantly high heterogeneity observed in the pooled datasets. To better understand these interactions, the datasets were subgrouped based on the specific *Schistosoma* species: *S. haematobium*, *S. japonicum*, *S. mansoni*, *S. intercalatum*, and *S. haematobium*-*S. mansoni* coinfection. This subgrouping allowed for a detailed assessment of the associations between individual *Schistosoma* species and their interactions with the host microbiome. A random-effects model was applied to analyze these subgroups, which revealed significant heterogeneity between the groups (Q = 12.91, df = 4, *p* = 0.0117). The RR and 95% CI for each subgroup were as follows: *S. haematobium* (k = 18, RR (95% CI) = 1.4188 (0.9732–2.0683)), *S. japonicum* (k = 1, RR (95% CI) = 1.0763 (0.7844–1.4769)), *S. mansoni* (k = 5, RR (95% CI) = 1.6496 (0.7324–3.7156), *S. intercalatum* (k = 1, RR (95% CI) = 3.0170 (1.7758–5.1260), *S. haematobium-S. mansoni* coinfection ((k = 5, RR (95% CI) = 0.9421 (0.4257–2.0850)). The subgroup analysis demonstrated that *S. haematobium* was the most significant contributor to the overall heterogeneity, accounting for 62.1% (τ2 = 0.4962, Chi^2^ = 292.96, df = 17, *p* < 0.01; I^2^ = 94%), *S. mansoni* contributed 13.0% (τ^2^ = 0, Chi^2^ = 12.11, df = 4, *p* = 0.02; I^2^ = 94%), and the coinfection of *S. haematobium* and *S. mansoni* accounted for 16.8% of the heterogeneity (τ^2^ = 0.1094, Chi^2^ = 21.98, df = 4, *p* < 0.01; I^2^ = 82%). This indicates that *S. haematobium* and its coinfection with *S. mansoni* are major factors contributing to the variability seen in the pooled analysis (Figure 5). Similarly, the analysis of risk differences among subgroups also highlighted significant heterogeneity between groups (Q = 30.94, df = 4, *p* < 0.0001). The risk difference (RD) and 95% confidence intervals for each subgroup were *S. haematobium* (k = 18, RD (95% CI) = 0.0571 (−0.0485–0.1626)), *S. japonicum* (k = 1, RD (95% CI) = 0.0376 (−0.1232–0.1984)), *S. mansoni* (k = 5, RD (95% CI) = 0.1547 (−0.1432–0.4527)), *S. intercalatum* (k = 1, RD (95% CI) = 0.4931 (0.3347–0.6514)), and *S. haematobium* and *S. mansoni* coinfection (k = 5, RD (95% CI) = −0.0263 (−0.1728–0.1202)) (Figure 6).

### 3.4. Influence of Praziquantel Treatment on Schistosomiasis Interactions with the Host Microbiome

**Figure 7:**
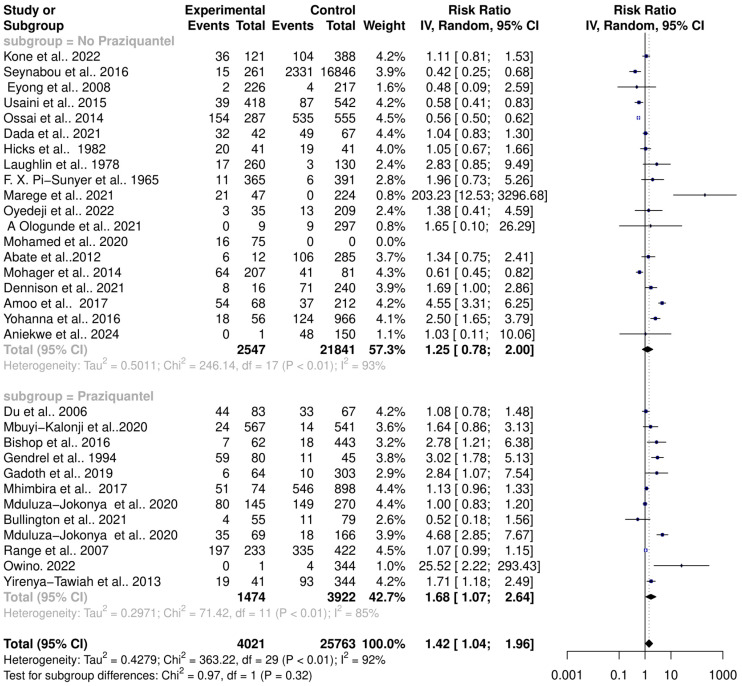
Forest plot showing the risk ratio of schistosomiasis and bacterial infection based on praziquantel treatment.

One of the cardinal side effects of praziquantel treatment is stomach aches and vomiting, suggesting possible disturbances of the intestinal microbiome. The effect of praziquantel treatment on schistosomiasis–host–microbiome interactions was assessed. The subgroup analysis showed that there is a significant risk change in the microbiome among *Schistosoma*-infected individuals in both praziquantel treatment (RR = 1.68, 95% CI = 1.07; 2.64) with high heterogeneity (Tau^2^ = 0.2971, Chi^2^ = 71.42, df = 11, *p* < 0.01); I^2^ = 85%) and non-praziquantel treatment (RR = 1.25, 95% CI = 0.78; 2.00) with high heterogeneity (Tau^2^ = 0.5011, Chi^2^ = 246.14, df = 17, *p* < 0.01); I^2^ = 93%). However, there was no significant difference among the subgroups (Chi^2^ = 0.91, df = 1, *p* = 0.32) (Figure 7).

**Figure 8:**
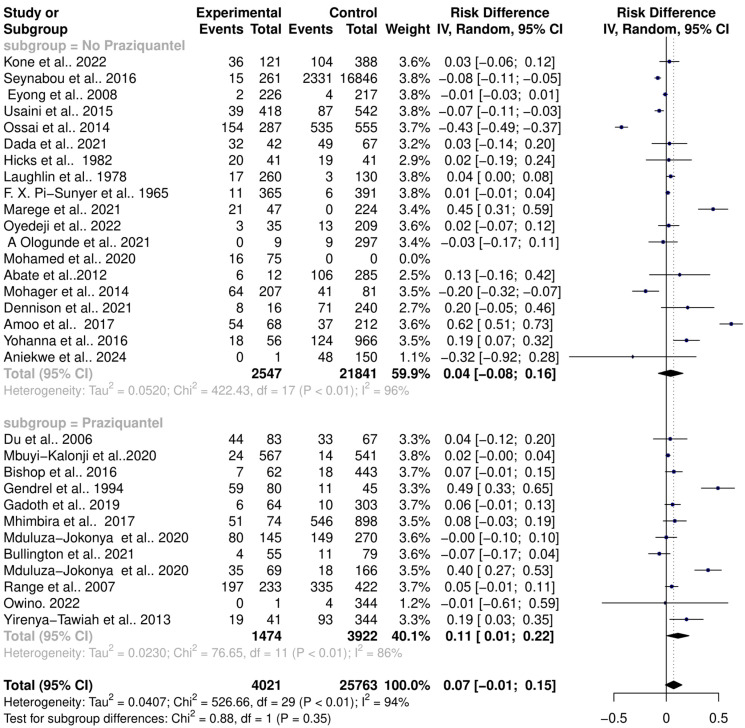
Forest plot showing the risk difference of schistosomiasis and bacterial infection based on praziquantel treatment.

Similarly, there was no difference between the risk difference (RD) for praziquantel treatment (RD = 0.04, 95% CI = −0.06; 0.16) and non-praziquantel treatment (RD = 0.11, 95% CI = 0.01; 0.22) (Figure 8). This confirms that there is a significant risk of change in the microbiome among *Schistosoma*-infected individuals in both praziquantel treatment and non-praziquantel treatment. Interestingly, *H. pylori*, *Acinetobacter* spp., *Providencia* spp., *Chlamydia*, *Lactobacillus*, *Gardnerella*, *Megasphaera*, *Sneathia*, *Peptostreptococcus*, and *Prevotella* were uniquely isolated from praziquantel-treated individuals, while individuals without praziquantel treatment uniquely had *Enterococcus*, *Staphylococcus*, *Proteus*, *Moraxella* spp., *Streptococcus*, *Yersinia*, *Enterobacter*, *Morganella*, *Vibrio*, *Corynebacterium*, *Bacillus*, and *Kurthia* infections (Table 2).

### 3.5. Influence of Gender on Schistosomiasis Interactions with the Host Microbiome

**Figure 9:**
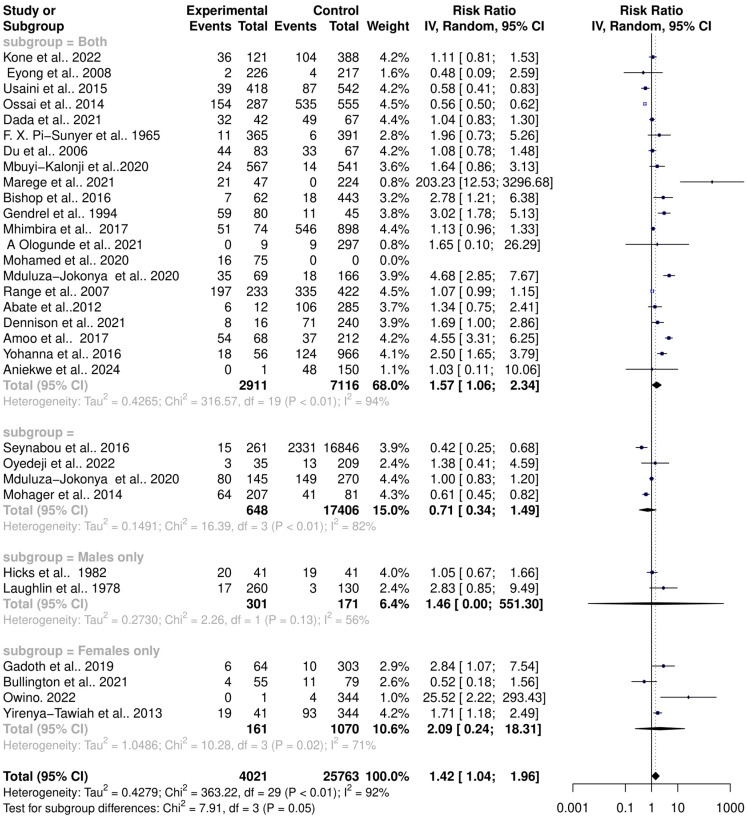
Forest plot showing the risk ratio of schistosomiasis and bacterial infection based on gender.

**Figure 10:**
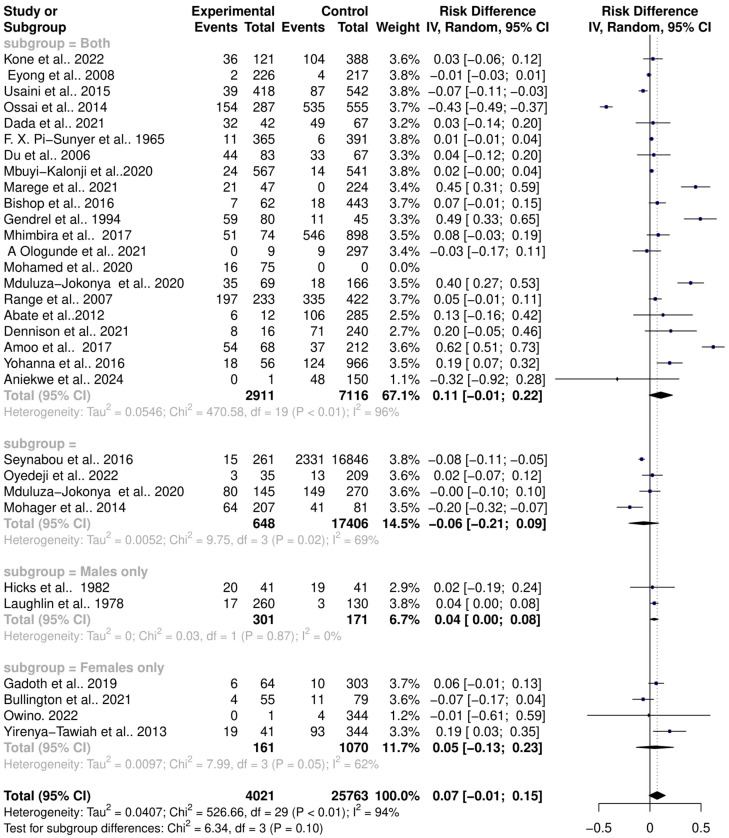
Forest plot showing the risk difference of schistosomiasis and bacterial infection based on gender.

Gender significantly impacts human microbiota diversity and composition due to anatomical differences, hormonal influences, immune system differences, and behavioral and lifestyle factors. Females typically have a vaginal microbiome dominated by *Lactobacillus* species, while males have a urethral microbiome influenced by skin and gut-associated bacteria. These gender-specific differences play an important role in schistosomiasis interactions with the host microbiome, such as bacterial vaginosis and urogenital infections. A gender subgroup analysis revealed that studies not differentiating between males and females showed a higher risk of *Schistosoma* infections altering the microbiota (RR = 1.57, 95% CI = 1.06; 2.34) with high heterogeneity (Tau^2^ = 0.4265, Chi^2^ = 316.57, df = 19, *p* < 0.01, I^2^ = 94%). Conversely, studies not reporting on gender recorded lower risks of schistosomiasis altering the host microbiota (RR = 0.71, 95% CI = 0.34; 1.49) with significant heterogeneity (Tau^2^ = 0.1491, Chi^2^ = 16.39, df = 3, *p* < 0.01, I^2^ = 82%). Further analysis showed that *Schistosoma* infections in males have a higher risk of altering the host microbiome (RR = 1.46, 95% CI = 0.00; 551.30) with high heterogeneity (Tau^2^ = 0.2730, Chi^2^ = 2.26, df = 1, *p* = 0.13, I^2^ = 56%). In females, the risk is even higher (RR = 2.09, 95% CI = 0.24; 18.31) with high heterogeneity (Tau^2^ = 1.0486, Chi^2^ = 10.28, df = 3, *p* = 0.02, I^2^ = 71%) (Figure 9). The risk difference (RD) for the random-effects model varied; studies not differentiating between genders showed RD = 0.11 (95% CI = −0.01; 0.22), studies not reporting on gender showed RD = −0.06 (95% CI = −0.21; 0.09), male-specific studies showed RD = 0.04 (95% CI = 0.00; 0.08), and female-specific studies showed RD = 0.05 (95% CI = −0.13; 0.23) (Figure 10). This indicates gender differences in microbiome diversity are altered by schistosomiasis.

In studies that did not differentiate between genders, the most commonly isolated microbes included *Enterococcus*, *Escherichia*, *Klebsiella*, *Pseudomonas*, *Staphylococcus*, *Serratia*, *Proteus*, *Yersinia*, *Salmonella*, *H. pylori*, *Citrobacter*, *Acinetobacter*, *Providencia*, *Mycobacterium*, and *Streptococcus*. In studies that did not report gender, the frequently isolated microbes were *Salmonella*, *Citrobacter*, *Enterobacter*, *Morganella*, *Vibrio*, *Escherichia coli*, *Neisseria species*, *Streptococcus*, *Corynebacterium*, *Bacillus*, *Kurthia*, *Enterococcus*, *Staphylococcus*, *Klebsiella* spp., *Pseudomonas* spp., and *Moraxella* spp. Male-specific studies reported *Staphylococcus* spp., *Proteus* spp., *Klebsiella*, *Escherichia coli*, *Streptococcus* spp., and *Pseudomonas* as frequently isolated microbes. In female-specific studies, the commonly isolated microbes were *Chlamydia*, *Neisseria*, *Lactobacillus*, *Gardnerella*, *Megasphaera*, *Sneathia*, *Peptostreptococcus*, and *Prevotella* (Table 2). These findings underscore the significant influence of gender on the interactions between schistosomiasis and the host microbiome, highlighting the necessity for gender-specific considerations in research and treatment strategies.

### 3.6. Meta-Regression Analysis for Schistosomiasis–Host Microbiome Interactions

**Figure 11:**
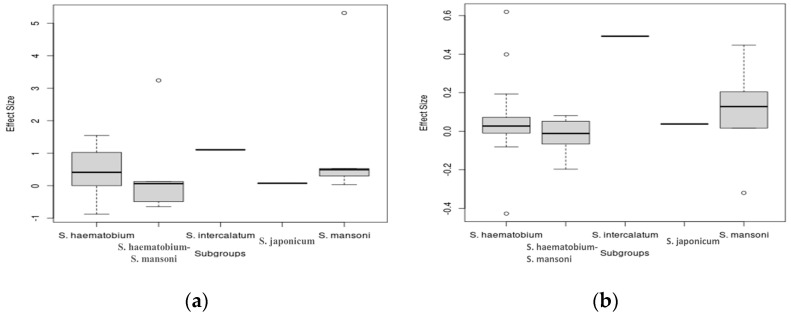
Boxplot showing the effect size distribution of *Schistosoma* species among schistosomiasis and bacterial coinfection; (**a**) effect size calculation from risk ratio, (**b**) effect size calculation from risk difference.

The meta-regression analysis, using a mixed-effects model with restricted maximum likelihood (REML) applied to 30 out of 31 studies (*k* = 30), explored the residual heterogeneity in the context of schistosomiasis–host microbiome interactions. The analysis revealed significant residual heterogeneity, with an estimated *τ*^2^ of 0.4587 (SE = 0.1679) and τ of 0.6773, indicating substantial unexplained variability in the interactions. The ratio of residual heterogeneity to unaccounted variability (*I*^2^) was extremely high at 94.38%, suggesting that the majority of variability among studies remains unexplained by the current model. Furthermore, the unaccounted variability to sampling variability ratio (*H*^2^) was 17.8, reflecting significant between-study variability. The model’s percentage of accounted heterogeneity (*R*^2^) was 0.00%, highlighting that the variability could not be adequately explained by the existing model. The heterogeneity test (QE) yielded a highly significant result (*df* = 25, *QE* = 327.0448, *p* < 0.0001), further underscoring the presence of considerable heterogeneity. The test of moderators indicated no significant interaction effect (*F* (*df*1 = 4, *df*2 = 25) = 0.5759, *p* = 0.6827), suggesting that specific interactions between schistosomiasis and the microbiome, influenced by *Schistosoma* species, are complex and not sufficiently explained by the model. The regression coefficient for the interaction between *Schistosoma* species and the microbiome, compared to *S. haematobium*, was 0.3482 (SE = 0.2055), with a 95% confidence interval of −0.0750 to 0.7714, indicating a 34.82% increase in the log relative risk of *Schistosoma* infection affecting the host microbiome (t = 1.6944, df = 25, *p* = 0.1026) (Figure 11a), although this increase was not statistically significant. Comparatively, *S. haematobium* and *S. mansoni* coinfections and microbiome interactions showed a 33.01% decrease relative to *S. haematobium* infections, while *S. japonicum* showed a 27.46% decrease. In contrast, *S. intercalatum* and *S. mansoni* demonstrated increases of 75.61% and 36.10%, respectively. The risk difference analysis indicated that only a small portion of the variability in interactions between schistosomiasis and the microbiome could be explained by *Schistosoma* species, with an R^2^ of 7.66%. The heterogeneity test (QE) again yielded a highly significant result (df = 28, QE = 478.4625, *p* < 0.0001), suggesting that both microbiome diversity and *Schistosoma* species contribute to variations in interactions. Additionally, the regression coefficient for *Schistosoma* species infection affecting the host microbiome was 0.0566 (SE = 0.0468), with a 95% confidence interval of −0.0398 to 0.1531, indicating a 5.66% increase in the log risk difference of *Schistosoma* species infection affecting the host microbiome (Figure 11b), highlighting the influence of *Schistosoma* species. This analysis underscores the complexity and significant interactions between Schistosoma species and the host microbiome, revealing high levels of residual heterogeneity and unaccounted variability.

### 3.7. Result of the Publication Bias Analysis 

**Figure 12:**
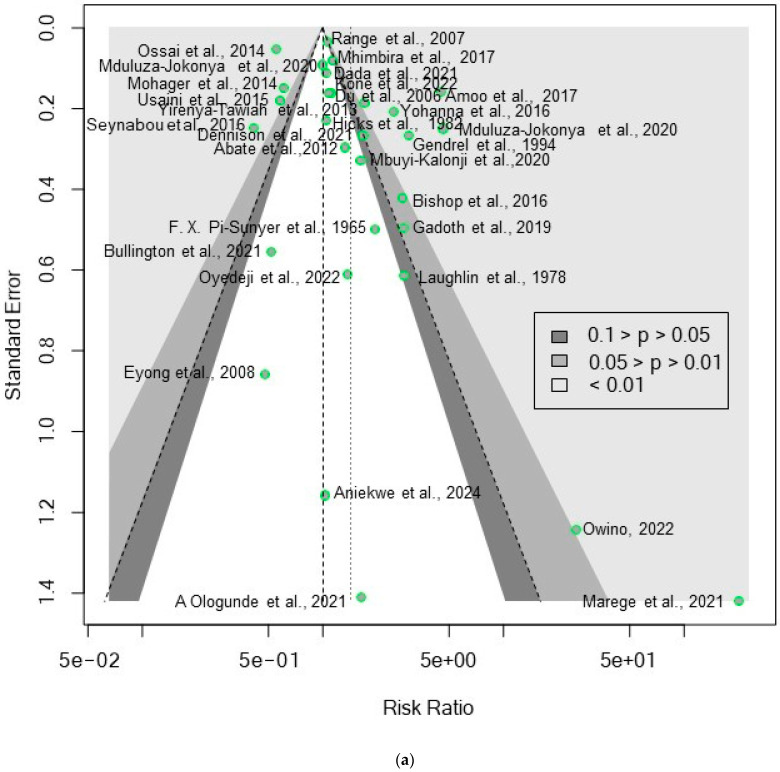

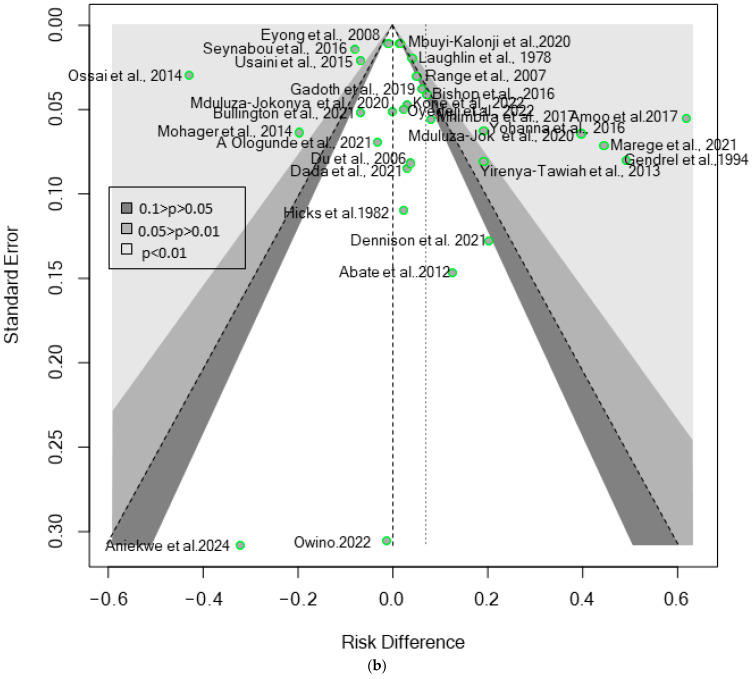
Funnel plot (Trim and Fill) showing asymmetrical distribution for schistosomiasis and bacterial infection from the 31 studies; (**a**) assess publication bias based on risk ratio analysis, (**b**) assess publication bias based on risk difference analysis.

The assessment of publication bias in the study revealed significant findings through multiple analytical approaches, including Fail-safe N calculation using the Rosenthal Approach, funnel plot (Trim and Fill) analysis, and Egger’s regression analysis for funnel plot asymmetry. The Fail-safe N calculation indicated a significant observed level of *p* < 0.0001, suggesting that it would require 271 additional unpublished studies with null results to render the overall effect non-significant at *p* = 0.05, implying robustness against publication bias. The funnel plot analysis (Trim and Fill) graphically displayed studies without apparent publication bias, suggesting a symmetrical distribution around the mean effect size. However, Egger’s regression analysis for funnel plot asymmetry showed mixed results; for the log risk ratio, a significant publication bias was confirmed (t = 2.19, df = 28, *p* = 0.0371), with a bias estimate (se. bias) of 1.8485 (0.8445) and a residual heterogeneity variance (τ^2^ = 11.0770) (Figure 12a). In contrast, the analysis for the log risk difference indicated no significant publication bias (t = 1.42, df = 28, *p* = 0.1654), with a bias estimate (se. bias) of 1.6048 (1.1269) and a residual heterogeneity variance (τ^2^ = 17.5387) (Figure 12b). The observed high heterogeneity in the pooled datasets suggests that factors such as the host-microbiome composition, host nutritional status, and environmental variables, rather than just the *Schistosoma* species, may contribute to the variability in study outcomes.

**Figure 13:**
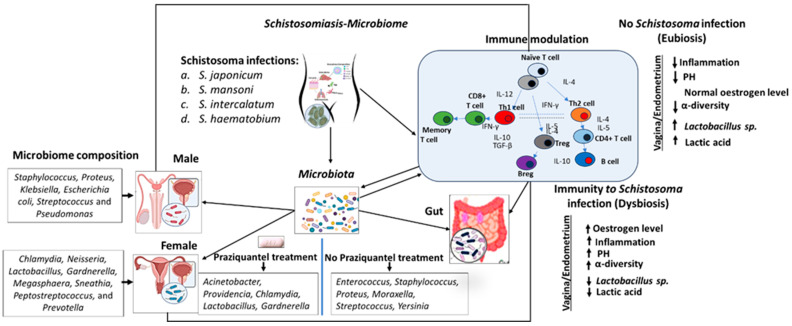
Interplay of schistosome infection, microbiome, and immune system.

The progression and severity of schistosomiasis are shaped by a complex interplay between *Schistosoma* infection, alterations in the host immune system, and changes in the host microbiome. When the parasite infects the host, it triggers an immune response that evolves from an initial Th1-type (pro-inflammatory) response to a chronic Th2-type response, which aims to limit tissue damage but can also cause fibrosis, especially in the liver. *Schistosoma* further manipulates the immune system by inducing regulatory T cells that suppress immune activity, aiding its persistence. Simultaneously, the infection alters the composition of the host’s gut microbiome, leading to changes that can either amplify or reduce inflammation through interactions between the microbiome and immune cells. These shifts can worsen the immune-mediated damage caused by the parasite’s eggs, affecting disease severity. This intricate relationship offers opportunities for targeted therapies, such as microbiome modulation or immune regulation, to better manage schistosomiasis (Figure 13).

## 4. Discussion

Schistosome infection disrupts the gut microbiota, leading to the colonization of pathogenic bacteria like *Salmonella* and *Helicobacter pylori*, causing severe disease outcomes [74,75]. It also creates a conducive environment for these bacteria to thrive, causing chronic UTIs and other organs’ pathogenesis [76]. Schistosome parasites manipulate the host’s immune system, forming granulomas to neutralize their eggs [77]. The orchestrated immune response promotes a Th2 immune profile while concurrently suppressing inflammatory reactions, resulting in severe disease outcomes and fostering conditions conducive to the development of antibiotic resistance [78]. In the case of *S. haematobium*, there is a disruption of the delicate balance of the urinary microbiota, heightening susceptibility to infections and perpetuating chronic inflammatory states [79].

The schistosomes have evolved a remarkable ability to modulate the host immune response to their advantage by triggering a distinctive T-helper 2 (Th2) immune response, marked by heightened production of cytokines such as interleukin-4 (IL-4), IL-5, and IL-13 [5,80]. Unlike the Th1 response, which is typically associated with directly attacking to eliminate pathogens, the Th2 response orchestrated by schistosomes aids in tissue repair and limits the extent of damage inflicted by the parasites [9,81]. Again, the schistosome stimulates regulatory T cell (Treg) proliferation and activation, which suppresses immune cell pro-inflammatory responses [82]. Understanding the interaction between schistosomiasis and the host’s microbiota and its implications for microbial dysbiosis, immune regulation, bacterial coinfections, antibiotic resistance, and therapeutic interventions could provide valuable insights. This systematic review and meta-analysis explore the relationship between schistosomiasis and the host’s microbiota to provide insights into disease pathogenesis and management.

The study found a high risk of *Schistosoma* infections disrupting the host’s microbiota, with no significant risk difference across all species, indicating microbial dysbiosis. The host microbiota alterations are significantly influenced by *S. haematobium*, *S. mansoni*, and coinfection of the *S. haematobium* and *S. mansoni*. The high heterogeneity across subgroups indicates significant variability in individual *Schistosoma* species and their interactions with the host microbiome.

Studies have shown that *S. haematobium* and *S. mansoni* infections lead to more diverse bacterial communities in the cervicovaginal area, urinary tract, and intestines due to immune environment changes and tissue damage [61,83]. Urinary schistosomiasis is frequently associated with bacteriuria, with *E. coli* being the most prevalent species [69,71,84].

In this study, 12 out of 31 studies (38.7%) reported *E. coli* isolation from schistosomiasis infections [43,44,45,47,48,49,50,51,55,56,70,71]. *E. coli* strains producing verotoxins (VT) are responsible for severe gastrointestinal diseases in humans, including hemolytic-uremic syndrome (HUS) [85]. A study isolated seven out of ten toxic strains from diarrhea-infected infants [86]. Verotoxigenic *E. coli* strains, notably O157, are globally dominant and frequently linked to severe outcomes like hemorrhagic colitis, characterized by bloody diarrhea, and HUS, a condition that can lead to kidney failure and systemic complications [87]. The frequent interaction between *Schistosoma* infections and *E. coli* can complicate the pathogenicity of both infections, particularly in how *Schistosoma* parasites propagate through the environment via their eggs [88]. *E. coli*, particularly pathogenic strains like Verotoxigenic *E. coli* (VTEC), can cause severe gastrointestinal illnesses such as diarrhea, often associated with contaminated water and food sources [89]. The bacteria-induced diarrhea facilitates the excretion of *Schistosoma* eggs, which subsequently hatch into larvae that infect intermediate freshwater snails [3].

The Infectious Disease Society of America (IDSA) has identified *Staphylococcus*, *Klebsiella*, and *Pseudomonas* as pathogenic bacteria known for their rapid development of antibiotic resistance [90]. These bacteria are associated with a range of disease states affecting organs such as the lungs, liver, and bloodstream, often initially colonizing the gastrointestinal tract [91]. Similar to *E. coli*, *Staphylococcus*, *Klebsiella*, and *Pseudomonas*, *H. pylori* has been implicated in the development of conditions like peptic ulcers, inflammatory bowel disease (IBD), and colorectal cancer (CRC) [92]. While *H. pylori* is traditionally associated with gastritis, peptic ulcer disease (PUD), gastric adenocarcinoma, and gastric mucosa-associated lymphoid tissue (MALT) lymphoma, it can also lead to upper respiratory tract infections (URTIs) [93]. Recent studies have highlighted the emergence of antibiotic-resistant strains of *Staphylococcus*, *Klebsiella*, and *Pseudomonas* in the intestinal tract following treatment for lung infections caused by these bacteria [94]. In Taiwan, China, research on genotoxic *Klebsiella pneumoniae* has demonstrated an increased prevalence among patients with pyogenic liver abscess (PLA), which has been linked to the development of CRC [95]. Despite these findings, the precise mechanisms by which *Staphylococcus*, *Klebsiella*, and *Pseudomonas* contribute to gastrointestinal (GIT) disease initiation and progression remain unclear.

In this study, it was found that eleven out of thirty-one articles (35.5%) reported the isolation of *Klebsiella* species [43,44,45,47,48,49,50,51,55,70,71], eight out of thirty-one articles (25.8%) reported the isolation of *Staphylococcus* species [43,47,48,49,56,70,71], while six out of thirty-one articles (19.4%) reported the isolation of *Pseudomonas* species [43,44,48,49,55,70] from individuals infected with *Schistosoma* species. This suggests a significant interaction between *Schistosoma* parasites and these bacterial species, akin to mechanisms observed with *E. coli*. *Schistosoma* species are known to interact with the host’s microbiota and immune system in ways that can facilitate the colonization and growth of secondary bacterial infections [30,96]. Similar to *E. coli*, these bacteria, including *Klebsiella*, *Staphylococcus*, and *Pseudomonas*, may exploit the altered immune environment and tissue damage caused by *Schistosoma* infections to establish themselves within the host [97]. The presence of these bacteria in individuals infected with *Schistosoma* underscores the complexity of host–parasite interactions and the potential for synergistic effects in disease pathogenesis and *Schistosoma* transmission.

*Proteus* infections can lead to various clinical manifestations, such as urethritis, cystitis, prostatitis, and pyelonephritis [98]. A persistent *Proteus* infection is often indicated by recurrent nephrolithiasis (kidney stone formation), as the history of a stone formation may suggest an ongoing or chronic infection [99,100]. *Proteus mirabilis* is known to instigate UTIs and catheter-associated urinary tract infections (CAUTIs), which can exacerbate conditions like urolithiasis (stone formation) in both the bladder and kidneys [101]. Coinfection with *Proteus mirabilis* and *Schistosoma* species can significantly complicate disease management and exacerbate clinical outcomes. This bacterium, a common cause of UTIs and pyelonephritis, thrives in environments altered by schistosomiasis, potentially leading to recurrent infections and chronic conditions [102]. In this study, five out of thirty-one articles (16.1%) reported the isolation of *Proteus* species from infections caused by *S. haematobium* [43,48,49,50,70]. This finding highlights the notable occurrence of *Proteus* bacteria, particularly *Proteus mirabilis*, in individuals infected with *S. haematobium*. In a co-infected scenario, the damage and inflammation from *Schistosoma* parasites can exacerbate the formation of kidney stones, creating a cycle of infection and stone recurrence.

*Streptococcus agalactiae*, known as Group B *Streptococcus* (GBS), is a common cause of 2–3% of UTIs in the human gastrointestinal and genitourinary tracts [103]. Its pathogenic mechanisms include surface-expressed protein adhesin molecules, immune-evasion factors, and toxins [104]. The bacterium can bind to bladder urothelial cells, stimulate IL-1α production, and induce inflammation [105]. Coinfection with *Streptococcus* spp. and *S. haematobium* complicates disease progression in the genitourinary system and treatment [106]. *S. haematobium* causes inflammation and tissue damage, attracting secondary infections like *S. agalactiae* [107]. This combination leads to severe symptoms and an increased risk of complications, and overlapping symptoms complicate diagnosis and treatment.

A hematogenous *Salmonella* UTI is a severe form of urinary tract infection that occurs when *Salmonella* bacteria spread through the bloodstream to the urinary system, rather than ascending directly from the lower urinary tract [108]. *Salmonella* infections colonize the gastrointestinal tract and enter the bloodstream, and colonization within macrophages facilitates the spread to infect organs like the liver, spleen, kidneys, and urinary tract [109,110]. This type of infection is less common but can occur in individuals with predisposing factors [111]. In eight out of thirty-one articles (25.8%) used in this study, the isolation of *Salmonella* from *Schistosoma* infections was reported; three out of the eight *Salmonella* spp. each were isolates from *S. haematobium* infections and *S. mansoni*, and one out of eight was a mixed infection of both *S. haematobium* and *S. mansoni* (12.5%) [48,51,53,56,57,63,67]. In a case of *Schistosoma intercalatum* infection, *Salmonella* spp. was isolated from an individual hospitalized with septicemic salmonellosis, suggesting a relapse of enteric fever triggered by *S. intercalatum*. [57]. The case of *S. intercalatum* infection concurrent with *Salmonella* spp. causing septicemic salmonellosis illustrates a complex interaction between chronic parasitic infection and bacterial disease [56]. *Schistosoma intercalatum*, primarily affecting the intestines, can induce chronic inflammation and immune modulation in infected individuals [112,113]. This immunological alteration may predispose patients to heightened susceptibility or severity of bacteria and parasitic infections [88]. Additionally, the intestinal damage and changes in gut microbiota caused by *Schistosoma* infection can create a conducive environment for the persistence and exacerbation of bacterial pathogens [114,115]. Hospitalization for septicemic salmonellosis underscores the severity of the bacterial infection, often exacerbated by underlying health conditions or immune compromise induced by chronic schistosomiasis [57].

Several studies have reported intriguing associations between *S. mansoni* infection and bacterial pathogens such as *Mycobacterium tuberculosis* [59,65,66] and *Mycobacterium leprae* [68]. Specifically, three studies documented a correlation between *S. mansoni* and *M. tuberculosis*, suggesting that chronic schistosomiasis may alter immune responses in a manner that affects susceptibility to tuberculosis or modifies its clinical course [59,65,66]. Concurrently, one study noted an association between *S. mansoni* and *M. leprae*, although the link was indirectly attributed to vitamin D deficiency, which can compromise immune function and potentially exacerbate susceptibility to both parasitic and bacterial infections [67].

It is interesting to note that various bacterial species, including *H. pylori*, *Acinetobacter*, *Bacillus*, *Chlamydia*, *Citrobacter*, *Corynebacterium*, *Enterobacter*, *Moraxella*, *Kurthia*, *Megasphaera*, and others, have been sporadically isolated from cases of *Schistosoma* infection [43,44,45,46,47,48,49,50,51,52,53,54,55,56,57,58,59,60,61,62,63,64,65,66,67,68,69,70,71,72,73]. The presence of schistosomes can both contribute to the onset of certain bacterial infections and exacerbate existing ones due to their impact on the host’s immune system. Schistosomiasis can alter immune responses, leading to a state of chronic inflammation and immunosuppression, which can make the host more susceptible to secondary infections. Additionally, the parasite-induced shifts in the gut microbiota can create an environment conducive to the growth of pathogenic bacteria. Therefore, while some infections may directly result from the immune alterations caused by schistosome presence, others could pre-exist but become more severe due to weakened immunity and changes in the microbial environment caused by schistosomiasis. This diverse range of bacteria suggests that individuals afflicted with schistosomiasis may harbor concurrent bacterial infections [116,117]. The presence of these bacteria could stem from various factors, such as compromised immune responses due to chronic parasitic infection, environmental exposures, or coinfections facilitated by shared transmission routes or habitats [118,119].

Schistosomiasis, whether treated with praziquantel or not, is associated with altered microbiota [26,120,121]. However, praziquantel-treated individuals have different microbiome compositions compared to individuals with no praziquantel treatment [122,123]. Notably, *Helicobacter pylori*, *Providencia* spp., *Chlamydia* spp., *Gardnerella vaginalis.*, *Megasphaera*, *Sneathia*, *Prevotella*, *Peptostreptococcus*, and *Lactobacillus* were the unique bacteria reported in praziquantel treatment cases. *Helicobacter pylori* is linked to peptic ulcers, chronic gastritis, and increased stomach cancer risk, while *Acinetobacter* spp. cause hospital-acquired infections like pneumonia, bacteremia, UTIs, and wound infections [124]. *Providencia* spp. leads to UTIs and bacteremia; *Chlamydia* species cause STIs, respiratory infections, and psittacosis; and *Gardnerella vaginalis*, *Megasphaera*, *Sneathia*, and *Prevotella* are associated with bacterial vaginosis and reproductive tract infections [125,126]. *Peptostreptococcus* causes anaerobic infections and bacteremia, whereas *Lactobacillus*, though generally beneficial, can rarely cause bacteremia or endocarditis in immunocompromised patients undergoing praziquantel treatment for schistosomiasis [127]. In schistosomiasis cases without praziquantel treatment, pathogens such as *Enterococcus*, *Staphylococcus*, *Proteus*, *Moraxella*, *Streptococcus*, *Yersinia*, *Enterobacter*, *Morganella*, *Vibrio*, *Corynebacterium*, *Bacillus*, and *Kurthia* are isolated, causing a range of infections, including urinary tract infections, respiratory infections, skin infections, gastrointestinal diseases, and more severe conditions like endocarditis, bacteremia, and diphtheria [128,129]. The severity of diseases caused by these pathogens varies depending on the specific organism and the host’s health status. Praziquantel treatment for schistosomiasis has a unique impact on the host microbiota, helping restore balance by reducing dysbiosis and inflammation compared to non-praziquantel treatments. Its effectiveness in clearing adult worms directly reduces chronic immune activation, allowing beneficial gut bacteria to recover. In contrast, non-praziquantel treatments may not target the parasites as directly, leading to less pronounced changes in microbiota and immune recovery.

Infections with schistosomiasis significantly impact the host microbiome, with females exhibiting a higher risk of microbiome alterations compared to males or combined genders [26,130,131]. Females typically have a vaginal microbiome dominated by *Lactobacillus* species, while males possess a urethral microbiome influenced by skin and gut-associated bacteria [132,133]. These gender-specific differences are crucial in understanding schistosomiasis interactions with the microbiome, as schistosomiasis can disrupt the vaginal microbiome in females, leading to bacterial vaginosis and other urogenital infections.

FGS represents a significant health issue that intricately interacts with the vaginal microbiome [15]. Schistosomiasis, particularly caused by *S. haematobium*, affects various aspects of the female reproductive system, including the vaginal microbiome [134]. Females typically have a vaginal environment dominated by *Lactobacillus* species, which plays a protective role against infections and maintains a healthy pH balance [132,135]. When schistosomiasis infects females, it disrupts this delicate balance. The presence of *S. haematobium* eggs in the vaginal and cervical regions can alter the normal microbiome composition, leading to the proliferation of potentially pathogenic bacteria and the development of conditions such as bacterial vaginosis [97,136,137]. This disruption is characterized by a decrease in *Lactobacillus* species and an increase in other bacteria, such as *Gardnerella*, *Megasphaera*, and *Prevotella* [138,139]. The presence of these bacteria is often associated with increased inflammation, which can exacerbate symptoms and contribute to chronic health issues [140,141]. Additionally, specific bacteria isolated from the vagina and cervix in females with schistosomiasis include *Chlamydia*, *Neisseria*, *Lactobacillus*, *Gardnerella*, *Megasphaera*, *Sneathia*, *Peptostreptococcus*, and *Prevotella*. Each of these bacteria can influence the severity of FGS and may interact with schistosomiasis in complex ways, potentially leading to increased susceptibility to secondary infections and complications [25,142]. Understanding these interactions is crucial for several reasons. Firstly, the altered microbiome can lead to a higher risk of bacterial vaginosis and other urogenital infections, complicating the clinical management of FGS [25,143]. Secondly, the impact of schistosomiasis on the vaginal microbiome highlights the need for gender-specific approaches to treatment and prevention [144,145,146]. For effective management of FGS, it is essential to address both the parasitic infection and the resulting microbiome imbalances.

The analysis of host microbiota–schistosomiasis interactions across different genders identified variations in bacterial species composition, but it remains unclear whether these differences correspond directly to variations in pathogenicity levels between genders. While certain bacterial taxa may be more prevalent or abundant in males or females, the study does not conclusively determine whether these compositional differences contribute to differing levels of disease severity or immune response. Additional information is needed to clarify whether the observed shifts in bacterial populations are merely reflective of gender-specific microbiota profiles or if they actively influence the pathogenicity and immune responses during schistosomiasis. Further research could provide insight into whether these microbial shifts affect infection outcomes differently in males and females.

The interaction between schistosomiasis, the immune system, and the microbiome involves intricate processes that significantly influence the progression and severity of the disease, as shown in Figure 13. Upon infection, *Schistosoma* parasites (*S. japonicum*, *S. mansoni*, *S. intercalatum*, and *S. haematobium*) penetrate the skin and mature within the host, prompting the immune system to recognize these invaders and initiate a response involving both the innate and adaptive immunity [9]. This immune response includes the activation of macrophages, dendritic cells, and the production of antibodies by B cells, which are critical for controlling the infection [147]. Schistosomiasis is characterized by a robust Th2 (T-helper 2) immune response, marked by the production of cytokines such as IL-4, IL-5, and IL-13 [81]. This Th2 response is crucial for forming granulomas around parasite eggs, which helps to contain the infection. However, it also contributes to chronic inflammation and tissue damage, particularly in the liver, intestines, and urinary tract, due to granuloma formation and subsequent fibrosis [148].

The urinary tract and gut microbiomes, diverse communities of microorganisms residing in these regions, play essential roles in regulating immune responses. A healthy microbiome helps maintain immune homeostasis by interacting with immune cells in the endometrium, vagina, and gut mucosa, influencing the balance between pro-inflammatory cytokines (such as IFN-γ and TGF-β) and Th2 cytokines (IL-4, IL-5, and IL-13) [149]. A balanced immune system can mitigate excessive inflammatory responses and promote effective pathogen clearance [150,151]. However, the composition of the microbiome can modulate the immune response to schistosomiasis, with certain microbial communities enhancing or suppressing the Th2 response that is central to the disease’s pathology [152]. For example, anti-inflammatory bacteria in the gut or vagina may reduce the severity of schistosomiasis by curbing excessive immune responses that lead to tissue damage [26].

Schistosomiasis itself can disrupt the balance of microbial communities, leading to dysbiosis. The immune response to *Schistosoma* and the resulting inflammation can alter the microbiome, often reducing the abundance of beneficial bacteria and allowing opportunistic or pathogenic microbes to flourish [153]. For instance, specific bacterial species such as *Staphylococcus*, *Proteus*, *Klebsiella*, *Escherichia coli*, *Streptococcus*, and *Pseudomonas* have been observed in *Schistosoma*-infected males, while females often harbor *Chlamydia*, *Neisseria*, *Lactobacillus*, *Gardnerella*, *Megasphaera*, *Sneathia*, *Peptostreptococcus*, and *Prevotella* [97,154]. Treatment with praziquantel, the only drug currently effective for schistosomiasis, can also influence microbiota composition, with shifts in species like *Acinetobacter*, *Providencia*, *Chlamydia*, *Lactobacillus*, and *Gardnerella* compared to untreated individuals, where different species such as *Enterococcus*, *Staphylococcus*, *Proteus*, *Moraxella*, *Streptococcus*, and *Yersinia* predominate [28].

This dysbiosis can further influence immune regulation, potentially resulting in a more pronounced inflammatory response or impaired immune function [155]. Changes in the microbiome due to schistosomiasis can create a feedback loop with the immune system, where each component continuously influences the other, exacerbating inflammation or altering the efficacy of immune responses [96,156]. This complex relationship between the immune system, microbiome, and schistosomiasis represents a challenge for treatment strategies. By targeting the microbiome through approaches like probiotics, prebiotics, or dietary interventions, it may be possible to modulate the immune response to schistosomiasis, potentially reducing disease severity and improving patient outcomes [157]. In conclusion, this study underscores the profound impact of schistosomiasis on the host microbiota across multiple bodily sites, including the urinary tract, vaginal tract, gut, skin, and lungs, disrupting the delicate microbial balance. This dysregulation involves bacterial species such as *E. coli*, *Klebsiella*, *Proteus*, *Pseudomonas*, *Salmonella*, *Staphylococcus*, *Streptococcus*, and *Mycobacterium* species (*M. tuberculosis* and *M. leprae*), frequently associated with schistosomiasis. The resultant dysbiosis contributes to the increased production of toxic metabolites, fostering inflammatory damage, particularly in the liver, kidney, and lungs, which exacerbates hepatic lesions and facilitates the progression of disease pathogenesis.

The impact of schistosomiasis on the vaginal microbiome underscores the necessity for gender-specific approaches to control and prevent the disease induced by *Schistosoma* infection. Effective management of FGS requires addressing both the parasitic infection and the resulting microbiome imbalances [146]. Also, the schistosomiasis cases treated with praziquantel had different bacteria compositions compared to the cases without praziquantel treatment. This suggests that combining praziquantel treatment with probiotics could potentially decrease the disease severity caused by an altered microbiome (Figure 13).

Moving forward, microbiota-based probiotic modulation emerges as a promising therapeutic approach. Probiotic supplementation has demonstrated significant antiapoptotic and antioxidant effects, potentially ameliorating the inflammatory sequelae of schistosomiasis-induced dysbiosis. Further research into the precise mechanisms of probiotic action and their clinical efficacy is warranted to harness their full therapeutic potential in managing the complex pathology of schistosomiasis.

## Figures and Tables

**Table 1:** Demographic characteristics of the 31 eligible studies included in the quantitative meta-analysis of schistosomiasis-microbiota interaction.

Schistosoma Species	Type of Bacteria Present	Country	Number of Participants	Males	Females	Methodology	Samples Used for Bacteria Isolation	Samples Used for Schistosoma Identification	Reference
*S. haematobium*	*Enterococcus faecalis*, *Escherichia coli*, *Klebsiella pneumoniae*, *Pseudomonas aeruginosa*, *Staphylococcus aureus Staphylococcus saprophyticus Serratia*, and *Proteus*	Nigeria	509	263	246	Microscopy, bacteria culture and Gram staining	Urine	Urine	[43]
*S. haematobium*	*Escherichia coli*, *Klebsiella* spp., *Pseudomonas* spp., *Moraxella* spp., and *Streptococcus*	Senegal	17,107	Not stated	Not stated	Microscopy, bacteria culture, Gram staining, biochemical tests and antimicrobial susceptibility testing	Urine	Urine	[44]
*S. haematobium*	*Escherichia coli* and *Klebsiella* spp.	Nigeria	443	Not stated	Not stated	Urine microscopy and bacteria culture	Urine	Urine	[45]
*S. haematobium*	Not stated	Nigeria	960	306	654	Sample culture, Gram staining and biochemical tests, urinalysis	Urine	Urine	[46]
*S. haematobium*	*Escherichia coli*, *Staphylococcus aureus*, and *Klebsiella* species	Nigeria	842	416	426	Urine filtration and microscopy, urinalysis, urine culture	Urine	Urine	[47]
*S. haematobium*	*Klebsiella pneumonia*, *Proteus vulgaris*, *Escherichia coli*, *Yersinia enterocolitica*, *Staphylococcus aureus*, *Enterobacter aerogenes*, *Salmonella enterica*, and *Pseudomonas aeruginosa*	Nigeria	109	35	74	Urine sedimentation and microscopy, urine culture, antimicrobial susceptibility testing	Urine	Urine	[48]
*S. haematobium*	*Staphylococcus aureus*, hemolytic *Staphylococcus albus*, *Proteus mirabilis*, *Klebsiella*, *Escherichia coli*, *Streptococcus faecelis*, *S. viridiens*, *S. haemolyticus*, various strains of *staphylococci* and *Pseudomonas aeruginosa*.	Egypt	82	Not stated	Not stated	Urine filtration and microscopy, urine analysis, Urine culture, Gram staining, and biochemical tests.	Urine	Urine	[49]
*S. haematobium*	*Escherichia coli*, *Klebsiella* species, and *Proteus* species.	Egypt	390	167	223	Urine culture, biochemical tests	Urine	Urine	[50]
*S. haematobium*	*Escherichia* sp., *Klebsiella* sp., *Staphylococcus* sp., and *Salmonella typhi*	Nigeria	656	370	286	Sedimentation and microscopy, urine culture, intradermal tests, flocculation tests and fluorescent antibody tests	Urine	Urine	[51]
*S. japonicum*	*H. pylori*	China	150	79	71	Kato Katz and microscopy, ELISA,	Blood	Stool	[52]
*S. mansoni*	*Salmonella* sp.	Democratic Republic of Congo	1108	504	554	Kato Katz and microscopy, stool and blood cultures, biochemical tests and antibiotic susceptibility testing	Stool	Stool	[53]
*S. mansoni*	*Salmonella* sp.	Ethiopia	271	165	106	Wet mount and stool concentration (formalin–ether concentration technique), Widal test, stool culture and biochemical tests and antimicrobial susceptibility test	Stool	Stool	[54]
*S. haematobium*	*Citrobacter* spp., *E. coli*, *Klebsiella* spp., *Acinetobacter* spp., *Providencia* spp., *Pseudomonas*, *Serratia*	Nigeria	505	254	251	Sedimentation and microscopy, urine culture, biochemical tests	Urine and blood	Urine	[55]
*S. haematobium*	*Salmonella enterica*, serovar *Paratyphi*, *Citrobacter freundii*, *Enterobacter aerogenes*, *Morganella morgani*, *Vibrio mirabilis*, *Escherichia coli*, *Neisseria* species, *Streptococcus pneumoniae*, *Corynebacterium xerosis*, *Bacillus cereus*, *Kurthia gibsoni*, *Enterococcus faecalis*, *Staphylococcus aureus*, *Streptococcus mitis* and *Staphylococcus saprophyticus*.	Nigeria	244	Not stated	Not stated	Sedimentation and microscopy, urinalysis, urine culture, biochemical tests, PCR, antibiotic susceptibility test	Urine	Urine	[56]
*S. intercalatum*	*Salmonella* spp.	Gabon	125	68	57	ELISA, Blood culture, antimicrobial susceptibility test, Rectal biopsy	Blood	Rectal biopsy	[57]
*S. haematobium*	*Chlamydia trachomatis* and *Neisseria gonorrhoeae*	Democratic Republic of Congo	367	0	367	Urine filtration and microscopy, nucleic acid amplification test (NAAT),	Vaginal swab	Urine	[58]
*S. haematobium* and *S. mansoni*	*Mycobacterium tuberculosis*	Tanzania	972	585	387	Baermann, FLOTAC, Kato–Katz, point-of-care circulating cathodic antigen urine cassette test, urine filtration, bacterial culture, AFB sputum smear using Ziehl–Nielsen methods and d Gene Xpert MTB/RIF	Sputum	Blood, stool and urine	[59]
*S. haematobium*	Upper Respiratory Tract Infection (No specific bacteria stated)	Zimbabwe	415	Not stated	Not stated	Hematuria check, clinical examination, Maglumi 4000 chemiluminescence immunoassay analyzer (CLIA) and urine filtration.	Not stated	Stool and urine	[60]
*S. haematobium* and *S. mansoni*	*Lactobacillus*, *Gardnerella*, *Megasphaera*, and *Sneathia*, *Peptostreptococcus anaerobius*, *Prevotella timonesis*	Tanzania	134	0	134	Microscopy and CAA, PCR and sequencing	Cervical swab	Stool and urine	[61]
*S. haematobium*	No *Salmonella* sp. isolated	Nigeria	306	118	188	Solid Rapid diagnostic test kit, urine sedimentation and microscopy	Blood (Serology)	Urine	[62]
*S. mansoni*	*S. paratyphi* B and *S. typhi*	Sudan	75	29	46	Kato–Katz, stool culture, gram staining, biochemical tests and Widal test	Stool	Stool	[63]
*S. haematobium*	Upper Respiratory Tract Infection (No specific bacteria stated)	Zimbabwe	235	129	108	Clinical examination, hematuria examination using Uristix reagent strips, urine filtration method, and microscopy	Not stated	Urine	[64]
*S. haematobium* and *S. mansoni*	*Mycobacterium tuberculosis*	Tanzania	655	386	269	Sputum culture and microscopy, Kato–Katz, membrane filtration and microscopy.	Sputum	Stool and urine	[65]
*S. mansoni*	*Mycobacterium tuberculosis*	Ethiopia	295	181	114	Sputum smear and microscopy, Kato–Katz technique, ELISA	Sputum	Stool	[66]
*S. haematobium* and *S. mansoni*	*Salmonella* spp.	Saudi Arabia	288	Not stated	Not stated	Clinical examination, Kato thick smear technique, centrifugation and microscopy, urine and stool culture, Gram staining and biochemical tests	Stool and urine	Stool and urine	[67]
*S. mansoni*	*Mycobacterium leprae*	Brazil	256	123	133	Kato–Katz and Hoffman–Pons–Janer methods	Skin slit	Blood and stool	[68]
*S. haematobium* and *S. mansoni*	Not stated	Kenya	345	0	345	Urine centrifugation and microscopy, Kato–Katz technique	Stool and urine	Stool and urine	[69]
*S. haematobium*	*Staphylococcus aureus*, *Proteus* species, *Klebsiella* species, *Escherichia coli*, *Pseudomonas aeruginosa*, *Streptococcus* species	Nigeria	280	120	160	Urinalysis, urine microscopy, urine culture, biochemical tests and Gram staining	Urine	Urine	[70]
*S. haematobium*	*Escherichia coli*, *Staphylococcus aureus*, *Klebsiella* species	Nigeria	1024	352	672	Urine culture, biochemical tests	Urine	Urine	[71]
*S. haematobium*	Bacterial vaginosis (No specific bacteria stated)	Ghana	385	Not stated	Not stated	Compressed biopsy technique	Vaginal swab and blood	Cervical biopsy	[72]
*S. mansoni*	*H. pylori*	Nigeria	151	Not stated	Not stated	Stool antigen test and conventional PCR assay, formol-ether concentration and nested PCR assay	Stool	Stool	[73]

**Table 2:** Composition of the microbiota among gender and Praziquantel treatment cases.

Treatment	Specific Bacteria Species	Bacteria Found in Both Categories
Praziquantel	*H. pylori* [52], *Acinetobacter* spp. [55], *Providencia* spp. [55], *Chlamydia* [58], *Lactobacillus* [61], *Gardnerella* [61], *Megasphaera* [61], *Sneathia* [61], *Peptostreptococcus* [61], and *Prevotella* [61].	*Salmonella* [48,51,53,54,56,57,63,67], *Citrobacter* spp. [55,56], *Escherichia coli* [43,44,45,47,48,49,50,51,55,56,70,71], *Klebsiella* spp. [43,44,45,47,48,49,50,51,55,70,71], *Pseudomonas* [43,44,48,49,55,70], *Serratia* [43,55], *Neisseria* [56,58], *Mycobacterium* [59,65,66,68].
No praziquantel	*Enterococcus* [43,56], *Staphylococcus* [43,44,47,48,49,51,56,70,71], *Proteus* [43,48,49,50,70], *Moraxella* spp. [44], *Streptococcus* [44,49,56,70], *Yersinia* [48], *Enterobacter* [48,56], *Morganella* [56], *Vibrio* [56], *Corynebacterium* [56], *Bacillus* [56], *Kurthia* [56].	
Gender		
Male only	*Staphylococcus* species [49], *Proteus* species. [49,50], *Klebsiella* [49,50], *Escherichia coli* [49,50], *Streptococcus* species [49], and *Pseudomonas* [49].	*Enterococcus* [43], *Escherichia* [43,45,47,48,51,55,70,71], *Klebsiella* [43,45,47,48,51,55,70,71], *Pseudomonas* [43,48,55,70], *Staphylococcus* [43,47,48,51,70,71], *Serratia* [43,55], *Proteus* [43,48,70], *Yersinia* [48], *Salmonella* [48,51,53,54,57,63], *H. pylori* [52], *Citrobacter* [55], *Acinetobacter* [55], *Providencia* [55], *Mycobacterium* [59,65,66,68], *Streptococcus* [70], *Enterobacter* [48]
Female only	*Chlamydia* [58], *Neisseria* [58], *Lactobacillus* [61], *Gardnerella* [61], *Megasphaera* [61], *Sneathia* [61], *Peptostreptococcus* [61], and *Prevotella* [61].	
Not Stated	*Salmonella* [56,67], *Citrobacter* [56], *Enterobacter* [44,56], *Morganella* [56], *Vibrio* [56], *Escherichia coli* [44,56], Neisseria species [56], *Streptococcus* [44,56], *Corynebacterium* [56], *Bacillus* [56], *Kurthia* [56], *Enterococcus* [56], *Staphylococcus* [44,56], *Klebsiella* species [44], *Pseudomonas* spp. [44], and *Moraxella* spp. [44].	

## Supplementary Materials

The following supporting information can be downloaded at: https://www.mdpi.com/article/10.3390/pathogens13100906/s1, Table S1: PRISMA Checklist; Table S2: Literature Search Strategy; Table S3: Joanna Briggs Institute critical appraisal checklist guidelines for Quality assessment; Table S4: Quality assessment results.

## References

[1] World Health Organization (WHO) Schistosomiasis Fact Sheet 2021. https://www.who.int/news-room/fact-sheets/details/schistosomiasis.

[2] McManus D.P., Bergquist R., Cai P., Ranasinghe S., Tebeje B.M., You H. (2020). Schistosomiasis—From immunopathology to vaccines. Seminars in Immunopathology.

[3] Ponzo E., Midiri A., Manno A., Pastorello M., Biondo C., Mancuso G. (2024). Insights into the epidemiology, pathogenesis, and differential diagnosis of schistosomiasis. Eur. J. Microbiol. Immunol..

[4] Verjee M.A. (2019). Schistosomiasis: Still a cause of significant morbidity and mortality. Res. Rep. Trop. Med..

[5] Mu Y., McManus D.P., Hou N., Cai P. (2021). Schistosome infection and schistosome-derived products as modulators for the prevention and alleviation of immunological disorders. Front. Immunol..

[6] Dastoli P.A., da Costa M.D., Nicácio J.M., Pinho R.S., Ferrarini M.A., Cavalheiro S. (2023). Mansonic neuroschistosomiasis in the childhood: An undiagnosed pathology?. Child’s Nerv. Syst..

[7] Mawa P.A., Kincaid-Smith J., Tukahebwa E.M., Webster J.P., Wilson S. (2021). Schistosomiasis morbidity hotspots: Roles of the human host, the parasite and their interface in the development of severe morbidity. Front. Immunol..

[8] Schwartz C., Fallon P.G. (2018). Schistosoma “eggs-iting” the host: Granuloma formation and egg excretion. Front. Immunol..

[9] Costain A.H., MacDonald A.S., Smits H.H. (2018). Schistosome egg migration: Mechanisms, pathogenesis and host immune responses. Front. Immunol..

[10] Takaki K.K., Rinaldi G., Berriman M., Pagán A.J., Ramakrishnan L. (2021). *Schistosoma mansoni* eggs modulate the timing of granuloma formation to promote transmission. Cell Host Microbe.

[11] Giorgio S., Gallo-Francisco P.H., Roque G.A., Flóro e Silva M. (2020). Granulomas in parasitic diseases: The good and the bad. Parasitol. Res..

[12] Ruff W.E., Greiling T.M., Kriegel M.A. (2020). Host–microbiota interactions in immune-mediated diseases. Nat. Rev. Microbiol..

[13] Wang C., Li Q., Ren J. (2019). Microbiota-immune interaction in the pathogenesis of gut-derived infection. Front. Immunol..

[14] Ogongo P., Nyakundi R.K., Chege G.K., Ochola L. (2022). The road to elimination: Current state of schistosomiasis research and progress towards the end game. Front. Immunol..

[15] Rossi B., Previtali L., Salvi M., Gerami R., Tomasoni L.R., Quiros-Roldan E. (2024). Female Genital Schistosomiasis: A Neglected among the Neglected Tropical Diseases. Microorganisms.

[16] Bustinduy A.L., Randriansolo B., Sturt A.S., Kayuni S.A., Leutscher P.D., Webster B.L., Van Lieshout L., Stothard J.R., Feldmeier H., Gyapong M. (2022). An update on female and male genital schistosomiasis and a call to integrate efforts to escalate diagnosis, treatment and awareness in endemic and non-endemic settings: The time is now. Adv. Parasitol..

[17] Sadeghpour Heravi F. (2024). Host-vaginal microbiota interaction: Shaping the vaginal microenvironment and bacterial vaginosis. Curr. Clin. Microbiol. Rep..

[18] Lamberti O., Bozzani F., Kiyoshi K., Bustinduy A.L. (2024). Time to bring female genital schistosomiasis out of neglect. Br. Med. Bull..

[19] Moeller A.H., Sanders J.G. (2020). Roles of the gut microbiota in the adaptive evolution of mammalian species. Philos. Trans. R. Soc. B.

[20] Hooper L.V., Macpherson A.J. (2010). Immune adaptations that maintain homeostasis with the intestinal microbiota. Nat. Rev. Immunol..

[21] Thakur A., Mikkelsen H., Jungersen G. (2019). Intracellular pathogens: Host immunity and microbial persistence strategies. J. Immunol. Res..

[22] Soares M.P., Teixeira L., Moita L.F. (2017). Disease tolerance and immunity in host protection against infection. Nat. Rev. Immunol..

[23] Floudas A., Aviello G., Schwartz C., Jeffery I.B., O’Toole P.W., Fallon P.G. (2019). *Schistosoma mansoni* worm infection regulates the intestinal microbiota and susceptibility to colitis. Infect. Immun..

[24] Douglas B., Oyesola O., Cooper M.M., Posey A., Tait Wojno E., Giacomin P.R., Herbert D.B. (2021). Immune system investigation using parasitic helminths. Annu. Rev. Immunol..

[25] Perera D.J., Koger-Pease C., Paulini K., Daoudi M., Ndao M. (2024). Beyond schistosomiasis: Unraveling co-infections and altered immunity. Clin. Microbiol. Rev..

[26] de Araújo M.P., Sato M.O., Sato M., Wm K.M., Coelho L.F., Souza R.L., Kawai S., Marques M.J. (2022). Unbalanced relationships: Insights into the interaction between gut microbiota, geohelminths, and schistosomiasis. PeerJ.

[27] Hong A., Umar A., Chen H., Yu Z., Huang J. (2024). Advances in the study of the interaction between schistosome infections and the host’s intestinal microorganisms. Parasites Vectors.

[28] Schneeberger P.H., Coulibaly J.T., Panic G., Daubenberger C., Gueuning M., Frey J.E., Keiser J. (2018). Investigations on the interplays between *Schistosoma mansoni*, praziquantel and the gut microbiome. Parasites Vectors.

[29] Sousa-Figueiredo J.C., Pleasant J., Day M., Betson M., Rollinson D., Montresor A., Kazibwe F., Kabatereine N.B., Stothard J.R. (2010). Treatment of intestinal schistosomiasis in Ugandan preschool children: Best diagnosis, treatment efficacy and side-effects, and an extended praziquantel dosing pole. Int. Health.

[30] Beyhan Y.E., Yıldız M.R. (2023). Microbiota and parasite relationship. Diagn. Microbiol. Infect. Dis..

[31] Yu L.C., Wang J.T., Wei S.C., Ni Y.H. (2012). Host-microbial interactions and regulation of intestinal epithelial barrier function: From physiology to pathology. World J. Gastrointest. Pathophysiol..

[32] Postler T.S., Ghosh S. (2017). Understanding the holobiont: How microbial metabolites affect human health and shape the immune system. Cell Metab..

[33] Reid G., Younes J.A., Van der Mei H.C., Gloor G.B., Knight R., Busscher H.J. (2011). Microbiota restoration: Natural and supplemented recovery of human microbial communities. Nat. Rev. Microbiol..

[34] Stevens E.J., Bates K.A., King K.C. (2021). Host microbiota can facilitate pathogen infection. PLoS Pathog..

[35] Hrncir T. (2022). Gut microbiota dysbiosis: Triggers, consequences, diagnostic and therapeutic options. Microorganisms.

[36] Saltzman E.T., Palacios T., Thomsen M., Vitetta L. (2018). Intestinal microbiome shifts, dysbiosis, inflammation, and non-alcoholic fatty liver disease. Front. Microbiol..

[37] Maggini S., Pierre A., Calder P.C. (2018). Immune function and micronutrient requirements change over the life course. Nutrients.

[38] Moreira-Filho J.T., Dantas R.F., Senger M.R., Silva A.C., Campos D.M., Muratov E., Silva-Junior F.P., Andrade C.H., Neves B.J. (2019). Shortcuts to schistosomiasis drug discovery: The state-of-the-art. Annual Reports in Medicinal Chemistry.

[39] Parums D.V. (2021). Review articles, systematic reviews, meta-analysis, and the updated preferred reporting items for systematic reviews and meta-analyses (PRISMA) 2020 guidelines. Med. Sci. Monit. Int. Med. J. Exp. Clin. Res..

[40] Migliavaca C.B., Stein C., Colpani V., Munn Z., Falavigna M. (2020). Quality assessment of prevalence studies: A systematic review. J. Clin. Epidemiol..

[41] Topçuoğlu M.A., Arsava E.M. (2023). Secondary Stroke Prevention in Patients with Patent Foramen Ovale: To Anticoagulate or Not? Fragility Index Meta-analysis of Published Randomized Controlled Studies. Turk. J. Neurol..

[42] Nakagawa S., Lagisz M., Jennions M.D., Koricheva J., Noble D.W., Parker T.H., Sánchez-Tójar A., Yang Y., O’Dea R.E. (2022). Methods for testing publication bias in ecological and evolutionary meta-analyses. Methods Ecol. Evol..

[43] Kone K.J., Onifade A.K., Dada E.O. (2022). Occurrence of urinary schistosomiasis and associated bacteria in parts of Ondo State, Nigeria. PLoS Glob. Public Health.

[44] Seynabou L., Awa B.D., Oumarou F.D., Moustapha M., Makhtar C., Mamadou D., Rokhaya D., Mamadou L.D., Roughyatou K., Babacar F. (2016). Profile of bacterial and parasitic urinary infections in Saint Louis Senegal between 2000 and 2010. Afr. J. Microbiol. Res..

[45] Eyong M.E., Ikepeme E.E., Ekanem E.E. (2008). Relationship between *Schistosoma haematobium* infection and urinary tract infection among children in South Eastern, Nigeria. Niger. Postgrad. Med. J..

[46] Sm A., Ha H., Ky H., Ta I. (2015). Studies on Seasonal Variations in the Occurrences of *Schistosoma haematobium* and Bacterial Urinary Infections among School Age Children in Kano, Nigeria. IOSR J. Pharm. Biol. Sci. (IOSR-JPBS).

[47] Ossai O.P., Dankoli R., Nwodo C., Tukur D., Nsubuga P., Ogbuabor D., Ekwueme O., Abonyi G., Ezeanolue E., Nguku P. (2014). Bacteriuria and urinary schistosomiasis in primary school children in rural communities in Enugu State, Nigeria, 2012. Pan Afr. Med. J..

[48] Dada E.O., Alagha B. (2021). Urinary schistosomiasis and asymptomatic bacteriuria among individuals of Ipogun, Nigeria: Detection of predominant microorganisms and antibiotic susceptibility profile. J. Med. Health Stud..

[49] Hicks R.M., Ismail M.M., Walters C.L., Beecham P.T., Rabie M.F., El Alamy M.A. (1982). Association of bacteriuria and urinary nitrosamine formation with *Schistosoma haematobium* infection in the Qalyub area of Egypt. Trans. R. Soc. Trop. Med. Hyg..

[50] Laughlin L.W., Farid Z., Mansour N., Edman D.C., Higashi G.I. (1978). Bacteriuria in urinary schistosomiasis in Egypt: A prevalence survey. Am. J. Trop. Med. Hyg..

[51] Pi-Sunyer F.X., Gilles H.M., Wilson A.M. (1965). *Schistosoma haematobium* infection in Nigeria: I.—Bacteriological and immunological findings in the presence of schistosomal infection. Ann. Trop. Med. Parasitol..

[52] Du Y., Agnew A., Ye X.P., Robinson P.A., Forman D., Crabtree J.E. (2006). Helicobacter pylori and *Schistosoma japonicum* co-infection in a Chinese population: Helminth infection alters humoral responses to H. pylori and serum pepsinogen I/II ratio. Microbes Infect..

[53] Mbuyi-Kalonji L., Barbé B., Nkoji G., Madinga J., Roucher C., Linsuke S., Hermy M., Heroes A.S., Mattheus W., Polman K. (2020). Non-typhoidal Salmonella intestinal carriage in a *Schistosoma mansoni* endemic community in a rural area of the Democratic Republic of Congo. PLoS Negl. Trop. Dis..

[54] Marege A., Seid M., Boke B., Thomas S., Arage M., Mouze N., Yohanes T., Woldemariam M., Manilal A. (2021). Prevalence of *Schistosoma mansoni*–Salmonella coinfection among patients in southern Ethiopia. New Microbes New Infect..

[55] Bishop H.G., Inabo H.I., Ella E.E. (2016). Salmonella-Bacteraemia and Diversity of Bacterial Uropathogens in Concomitant Urinary Schistosomiasis among Children in Jaba, Kaduna State, Nigeria. Int. J. Sci. Res. Environ. Sci..

[56] Oyedeji B.R., Idris O.O., Agunbiade B.T., Olabiyi O.E., Oluboyo B.O., Okiki P.A. (2022). Occurrence of Significant Bacteriuria Among Schistosomiasis Positive Individuals in Ekiti State, Nigeria. ABUAD Int. J. Nat. Appl. Sci..

[57] Gendrel D., Kombila M., Beaudoin-Leblevec G., Richard-Lenoble D. (1994). Nontyphoidal salmonellal septicemia in Gabonese children infected with *Schistosoma intercalatum*. Clin. Infect. Dis..

[58] Gadoth A., Mvumbi G., Hoff N.A., Musene K., Mukadi P., Ashbaugh H.R., Doshi R.H., Javanbakht M., Gorbach P., Okitolonda-Wemakoy E. (2019). Urogenital schistosomiasis and sexually transmitted coinfections among pregnant women in a schistosome-endemic region of the Democratic Republic of Congo. Am. J. Trop. Med. Hyg..

[59] Mhimbira F., Hella J., Said K., Kamwela L., Sasamalo M., Maroa T., Chiryamkubi M., Mhalu G., Schindler C., Reither K. (2017). Prevalence and clinical relevance of helminth co-infections among tuberculosis patients in urban Tanzania. PLoS Negl. Trop. Dis..

[60] Mduluza-Jokonya T.L., Naicker T., Jokonya L., Midzi H., Vengesai A., Kasambala M., Choto E., Rusakaniko S., Sibanda E., Mutapi F. (2020). Association of *Schistosoma haematobium* infection morbidity and severity on co-infections in pre-school age children living in a rural endemic area in Zimbabwe. BMC Public Health.

[61] Bullington B.W., Lee M.H., Mlingi J., Paul N., Aristide C., Fontana E., Littmann E.R., Mukerebe C., Shigella P., Kashangaki P. (2021). Cervicovaginal bacterial communities in reproductive-aged Tanzanian women with *Schistosoma mansoni*, *Schistosoma haematobium*, or without schistosome infection. ISME J..

[62] AOlogunde C., TAkinruli F., OLayo-Akingbade T. (2021). Malaria Co–Infection with Urinary Schistosomiasis, Typhoid Fever, Hepatitis B Virus, and Human Immunodeficiency (HIV) Virus among Students in Three Local Government Areas of Ekiti State, South Western Nigeria. Asian J. Res. Infect. Dis..

[63] Mohamed A.M., Hamad M.N. (2020). Relationship between intestinal Schistosomiasis and enteric fever among Sudanese patients, New Halfa Town, Kassala State, Sudan. J. Microbiol. Exp..

[64] Mduluza-Jokonya T.L., Vengesai A., Jokonya L., Thakataka A., Midzi H., Mduluza T., Sibanda E., Naicker T. (2020). Impact of Indolent Schistosomiasis on Morbidity and Mortality from Respiratory Tract Infections in Preschool Age Children from a Schistosomiasis Endemic Area. medRxiv.

[65] Range N., Magnussen P., Mugomela A., Malenganisho W., Changalucha J., Temu M.M., Mngara J., Krarup H., Friis H., Andersen Å.B. (2007). HIV and parasitic co-infections in tuberculosis patients: A cross-sectional study in Mwanza, Tanzania. Ann. Trop. Med. Parasitol..

[66] Abate E., Belayneh M., Gelaw A., Idh J., Getachew A., Alemu S., Diro E., Fikre N., Britton S., Elias D. (2012). The impact of asymptomatic helminth co-infection in patients with newly diagnosed tuberculosis in north-west Ethiopia. PLoS ONE.

[67] Mohager M.O., Mohager S.O., Kaddam L.A. (2014). The association between shistosomiasis and enteric fever in a single Schistosoma endemic area in Sudan. Int. J. Pharm. Sci. Res..

[68] Dennison C.L., de Oliveira L.B., Fraga L.A., e Lima R.S., Ferreira J.A., Clennon J.A., de Mondesert L., Stephens J., Magueta E.B., Branco A.C. (2021). Mycobacterium leprae–helminth co-infections and vitamin D deficiency as potential risk factors for leprosy: A case–control study in south-eastern Brazil. Int. J. Infect. Dis..

[69] Owino V.O. (2022). Prevalence and Risk Factors Associated with Urinary Schistosomiasis among Women of Reproductive Age in Nyando Sub-County, Kenya. Ph.D. Thesis.

[70] Amoo K.J., Amoo O.A., Oke A.A., Adegboyega T.T. (2017). Prevalence of urinary tract infection (UTI) and concomitant urinary schistosomiasis among primary school children in Remo north local government, Ogun state, Nigeria. IOSR J. Dent. Med. Sci..

[71] Yohanna J.A., Luka J.S., Dakul D.A., Bigila D.A., Akut F. (2016). Schisotosoma haematobium and Urinary Tract Infection (UTI) in Some Part of Jos, Plateau State, Nigeria. Int. J. Sci. Technoledge.

[72] Yirenya-Tawiah D.R., Amoah C.M., Apea-Kubi K.A., Dade M., Lomo G., Mensah D., Akyeh L., Bosompem K.M. (2013). Female genital schistosomiasis, genital tract infections and HIV co-infection in the Volta basin of Ghana. Int. J. Trop. Dis. Health.

[73] Aniekwe O., Jolaiya T., Ajayi A., Adeleye I.A., Gerhard M., Smith S.I. (2024). Co-infection of Helicobacter pylori and intestinal parasites in children of selected low-income communities in Lagos State, Nigeria. Parasitol. Int..

[74] Bajinka O., Qi M., Barrow A., Touray A.O., Yang L., Tan Y. (2022). Pathogenicity of Salmonella during Schistosoma-Salmonella Co-infections and the importance of the gut microbiota. Curr. Microbiol..

[75] Yang Y.J., Sheu B.S. (2016). Metabolic interaction of Helicobacter pylori infection and gut microbiota. Microorganisms.

[76] Lila A.S., Rajab A.A., Abdallah M.H., Rizvi S.M., Moin A., Khafagy E.S., Tabrez S., Hegazy W.A. (2023). Biofilm lifestyle in recurrent urinary tract infections. Life.

[77] Lundy S.K., Lukacs N.W. (2013). Chronic schistosome infection leads to modulation of granuloma formation and systemic immune suppression. Front. Immunol..

[78] Kayongo A., Robertson N.M., Siddharthan T., Ntayi M.L., Ndawula J.C., Sande O.J., Bagaya B.S., Kirenga B., Mayanja-Kizza H., Joloba M.L. (2023). Airway microbiome-immune crosstalk in chronic obstructive pulmonary disease. Front. Immunol..

[79] Azevedo M.M., Pina-Vaz C., Baltazar F. (2020). Microbes and cancer: Friends or faux?. Int. J. Mol. Sci..

[80] Acharya S., Da’dara A.A., Skelly P.J. (2021). Schistosome immunomodulators. PLoS Pathog..

[81] Masamba P., Kappo A.P. (2021). Immunological and biochemical interplay between cytokines, oxidative stress and schistosomiasis. Int. J. Mol. Sci..

[82] Tang C.L., Gao Y.R., Wang L.X., Zhu Y.W., Pan Q., Zhang R.H., Xiong Y. (2019). Role of regulatory T cells in Schistosoma-mediated protection against type 1 diabetes. Mol. Cell. Endocrinol..

[83] Sturt A.S., Webb E.L., Francis S.C., Hayes R.J., Bustinduy A.L. (2020). Beyond the barrier: Female genital schistosomiasis as a potential risk factor for HIV-1 acquisition. Acta Trop..

[84] Nwachukwu I.O., Ukaga C.N., Ajero C.M., Nwoke B.E., Nwachukwu M.I., Obasi C.C., Ezenwa C.M. (2018). Urinary Schistosomiasis and concomitant Bacteriuria among school age children in some parts of Owerri, Imo State. Int. Res. J. Adv. Eng. Sci..

[85] Piérard D., De Greve H., Haesebrouck F., Mainil J. (2012). O157: H7 and O104: H4 Vero/Shiga toxin-producing Escherichia coli outbreaks: Respective role of cattle and humans. Vet. Res..

[86] Ritchie J.M., Greenwich J.L., Davis B.M., Bronson R.T., Gebhart D., Williams S.R., Martin D., Scholl D., Waldor M.K. (2011). An Escherichia coli O157-specific engineered pyocin prevents and ameliorates infection by *E. coli* O157: H7 in an animal model of diarrheal disease. Antimicrob. Agents Chemother..

[87] Thomas R.R., Gaastra M.L., Brooks H.J. (2018). Shiga (Vero)-toxigenic’Escherichia coli’: Epidemiology, virulence and disease. N. Z. J. Med. Lab. Sci..

[88] Mabbott N.A. (2018). The influence of parasite infections on host immunity to co-infection with other pathogens. Front. Immunol..

[89] Chique C., Hynds P., Burke L.P., Morris D., Ryan M.P., O’Dwyer J. (2021). Contamination of domestic groundwater systems by verotoxigenic Escherichia coli (VTEC), 2003–2019: A global scoping review. Water Res..

[90] Tsuji B.T., Pogue J.M., Zavascki A.P., Paul M., Daikos G.L., Forrest A., Giacobbe D.R., Viscoli C., Giamarellou H., Karaiskos I. (2019). International consensus guidelines for the optimal use of the polymyxins: Endorsed by the American college of clinical pharmacy (ACCP), European society of clinical microbiology and infectious diseases (ESCMID), infectious diseases society of America (IDSA), international society for anti-infective pharmacology (ISAP), society of critical care medicine (SCCM), and society of infectious diseases pharmacists (SIDP). Pharmacother. J. Hum. Pharmacol. Drug Ther..

[91] Giuffrè M., Campigotto M., Campisciano G., Comar M., Crocè L.S. (2020). A story of liver and gut microbes: How does the intestinal flora affect liver disease? A review of the literature. Am. J. Physiol.-Gastrointest. Liver Physiol..

[92] Theresa M., Unni A.S., Geevarghese A., Sebastian S.K., Pareek S., Krishnankutty R.E. (2021). Foodborne Pathogens and Food-Related Microorganisms. Sequencing Technologies in Microbial Food Safety and Quality.

[93] Salena B.J., Hunt R.H., Sagar M., Padol I., Armstrong D., Moayyedi P., Yuan C., Marshall J. (1994). The stomach and duodenum. First Principles of Gastroenterology: The Basis of Disease and an Approach to Management.

[94] Terreni M., Taccani M., Pregnolato M. (2021). New antibiotics for multidrug-resistant bacterial strains: Latest research developments and future perspectives. Molecules.

[95] Lai Y.C., Lin A.C., Chiang M.K., Dai Y.H., Hsu C.C., Lu M.C., Liau C.Y., Chen Y.T. (2014). Genotoxic klebsiella pneumoniae in Taiwan. PLoS ONE.

[96] Cortés A., Toledo R., Cantacessi C. (2018). Classic models for new perspectives: Delving into helminth–microbiota–immune system interactions. Trends Parasitol..

[97] Ashour D.S., Othman A.A. (2020). Parasite–bacteria interrelationship. Parasitol. Res..

[98] Mancuso G., Midiri A., Gerace E., Marra M., Zummo S., Biondo C. (2023). Urinary tract infections: The current scenario and future prospects. Pathogens.

[99] Tamborino F., Cicchetti R., Mascitti M., Litterio G., Orsini A., Ferretti S., Basconi M., De Palma A., Ferro M., Marchioni M. (2024). Pathophysiology and Main Molecular Mechanisms of Urinary Stone Formation and Recurrence. Int. J. Mol. Sci..

[100] Espinosa-Ortiz E.J., Eisner B.H., Lange D., Gerlach R. (2019). Current insights into the mechanisms and management of infection stones. Nat. Rev. Urol..

[101] Yuan F., Huang Z., Yang T., Wang G., Li P., Yang B., Li J. (2021). Pathogenesis of Proteus mirabilis in catheter-associated urinary tract infections. Urol. Int..

[102] Le T.L., Sokolow S.H., Hammam O., Fu C.L., Hsieh M. (2015). Pathogenesis of human schistosomiasis. Human Emerging and Re-Emerging Infections: Viral and Parasitic Infections.

[103] Nguyen L.M., Omage J.I., Noble K., McNew K.L., Moore D.J., Aronoff D.M., Doster R.S. (2021). Group B streptococcal infection of the genitourinary tract in pregnant and non-pregnant patients with diabetes mellitus: An immunocompromised host or something more?. Am. J. Reprod. Immunol..

[104] Dash S., Duraivelan K., Samanta D. (2021). Cadherin-mediated host–pathogen interactions. Cell. Microbiol..

[105] Huang X., Pan T., Yan L., Jin T., Zhang R., Chen B., Feng J., Duan T., Xiang Y., Zhang M. (2021). The inflammatory microenvironment and the urinary microbiome in the initiation and progression of bladder cancer. Genes Dis..

[106] Uwandu C.U., Dike-Ndudim J.N., Ndubueze C.W. (2022). Epidemiological studies on urinary schistosomiasis and bacterial co-infection in some rural communities of Abia State, Nigeria. World J. Biol. Pharm. Health Sci..

[107] Çipe F., Arısoy E.S., Correa A.G. (2021). Immunological Responses to Infection. Pediatric ENT Infections.

[108] Arsene M.M., Viktorovna P.I., Davares A.K., Esther N., Nikolaevich S.A. (2021). Urinary tract infections: Virulence factors, resistance to antibiotics, and management of uropathogenic bacteria with medicinal plants: A review. J. Appl. Pharm. Sci..

[109] Foster N., Tang Y., Berchieri A., Geng S., Jiao X., Barrow P. (2021). Revisiting persistent Salmonella infection and the carrier state: What do we know?. Pathogens.

[110] Jajere S.M. (2019). A review of Salmonella enterica with particular focus on the pathogenicity and virulence factors, host specificity and antimicrobial resistance including multidrug resistance. Vet. World.

[111] Andino A., Hanning I. (2015). Salmonella enterica: Survival, colonization, and virulence differences among serovars. Sci. World J..

[112] Wiedemann M., Voehringer D. (2020). Immunomodulation and immune escape strategies of gastrointestinal helminths and schistosomes. Front. Immunol..

[113] Schramm G., Suwandi A., Galeev A., Sharma S., Braun J., Claes A.K., Braubach P., Grassl G.A. (2018). Schistosome eggs impair protective Th1/Th17 immune responses against Salmonella infection. Front. Immunol..

[114] Domenico B., Alice D.P., Lorenza L., La Torre G., Cocchiara R.A., Sestili C., Del Cimmuto A., La Torre G. (2022). The impact of environmental alterations on human microbiota and infectious diseases. Environmental Alteration Leads to Human Disease: A Planetary Health Approach.

[115] Wu Y., Duffey M., Alex S.E., Suarez-Reyes C., Clark E.H., Weatherhead J.E. (2022). The role of helminths in the development of non-communicable diseases. Front. Immunol..

[116] Schlosser-Brandenburg J., Midha A., Mugo R.M., Ndombi E.M., Gachara G., Njomo D., Rausch S., Hartmann S. (2023). Infection with soil-transmitted helminths and their impact on coinfections. Front. Parasitol..

[117] O’Ferrall A.M., Musaya J., Stothard J.R., Roberts A.P. (2024). Aligning antimicrobial resistance surveillance with schistosomiasis research: An interlinked One Health approach. Trans. R. Soc. Trop. Med. Hyg..

[118] Behringer D.C., Karvonen A., Bojko J. (2018). Parasite avoidance behaviours in aquatic environments. Philos. Trans. R. Soc. B Biol. Sci..

[119] Salkeld D., Hopkins S., Hayman D. (2023). Emerging Zoonotic and Wildlife Pathogens: Disease Ecology, Epidemiology, and Conservation.

[120] Kay G.L., Millard A., Sergeant M.J., Midzi N., Gwisai R., Mduluza T., Ivens A., Nausch N., Mutapi F., Pallen M. (2015). Differences in the faecal microbiome in *Schistosoma haematobium* infected children vs. uninfected children. PLoS Negl. Trop. Dis..

[121] Lin D., Song Q., Liu J., Chen F., Zhang Y., Wu Z., Sun X., Wu X. (2022). Potential gut microbiota features for non-invasive detection of schistosomiasis. Front. Immunol..

[122] Haraoui L.P., Blaser M.J. (2023). The Microbiome and Infectious Diseases. Clin. Infect. Dis..

[123] Minich J.J., Power C., Melanson M., Knight R., Webber C., Rough K., Bott N.J., Nowak B., Allen E.E. (2020). The southern bluefin tuna mucosal microbiome is influenced by husbandry method, net pen location, and anti-parasite treatment. Front. Microbiol..

[124] Doughari H.J., Ndakidemi P.A., Human I.S., Benade S. (2011). The ecology, biology and pathogenesis of *Acinetobacter* spp.: An overview. Microbes Environ..

[125] Moreland R.B., Choi B.I., Geaman W., Gonzalez C., Hochstedler-Kramer B.R., John J., Kaindl J., Kesav N., Lamichhane J., Lucio L. (2023). Beyond the usual suspects: Emerging uropathogens in the microbiome age. Front. Urol..

[126] Severgnini M., Morselli S., Camboni T., Ceccarani C., Salvo M., Zagonari S., Patuelli G., Pedna M.F., Sambri V., Foschi C. (2022). Gardnerella vaginalis clades in pregnancy: New insights into the interactions with the vaginal microbiome. PLoS ONE.

[127] Gonzalez G.A., Porto G., Tecce E., Oghli Y.S., Miao J., O’Leary M., Chadid D.P., Vo M., Harrop J. (2023). Advances in diagnosis and management of atypical spinal infections: A comprehensive review. N. Am. Spine Soc. J. (NASSJ).

[128] DellitTH O.R., McGowan J.E. (2018). Harrinarine Madhosingh, MD, FACP, FIDSA. Med. Secrets E-Book Med. Secrets E-Book.

[129] Dunachie S.J., Esmail H., Corrigan R., Dudareva M. (2022). Infectious Disease. Medicine for Finals and Beyond.

[130] Ajibola O., Rowan A.D., Ogedengbe C.O., Mshelia M.B., Cabral D.J., Eze A.A., Obaro S., Belenky P. (2019). Urogenital schistosomiasis is associated with signatures of microbiome dysbiosis in Nigerian adolescents. Sci. Rep..

[131] Ajibola O., Penumutchu S., Gulumbe B., Aminu U., Belenky P. (2023). Longitudinal analysis of the impacts of urogenital schistosomiasis on the gut microbiota of adolescents in Nigeria. Res. Sq..

[132] Lehtoranta L., Ala-Jaakkola R., Laitila A., Maukonen J. (2022). Healthy vaginal microbiota and influence of probiotics across the female life span. Front. Microbiol..

[133] Takada K., Melnikov V.G., Kobayashi R., Komine-Aizawa S., Tsuji N.M., Hayakawa S. (2023). Female reproductive tract-organ axes. Front. Immunol..

[134] Sturt A.S. (2021). The Cervicovaginal Environment and HIV Incidence in Zambian Women with Female Genital Schistosomiasis. Ph.D. Thesis.

[135] Chee W.J., Chew S.Y., Than L.T. (2020). Vaginal microbiota and the potential of Lactobacillus derivatives in maintaining vaginal health. Microb. Cell Factories.

[136] Adebayo A.S., Survayanshi M., Bhute S., Agunloye A.M., Isokpehi R.D., Anumudu C.I., Shouche Y.S. (2017). The microbiome in urogenital schistosomiasis and induced bladder pathologies. PLoS Negl. Trop. Dis..

[137] Colella M., Topi S., Palmirotta R., D’Agostino D., Charitos I.A., Lovero R., Santacroce L. (2023). An overview of the microbiota of the human urinary tract in health and disease: Current issues and perspectives. Life.

[138] Chen X., Lu Y., Chen T., Li R. (2021). The female vaginal microbiome in health and bacterial vaginosis. Front. Cell. Infect. Microbiol..

[139] Rosca A.S., Castro J., Sousa L.G., Cerca N. (2020). Gardnerella and vaginal health: The truth is out there. FEMS Microbiol. Rev..

[140] Ravel J., Moreno I., Simón C. (2021). Bacterial vaginosis and its association with infertility, endometritis, and pelvic inflammatory disease. Am. J. Obstet. Gynecol..

[141] Dabee S., Passmore J.A., Heffron R., Jaspan H.B. (2021). The complex link between the female genital microbiota, genital infections, and inflammation. Infect. Immun..

[142] Christinet V., Lazdins-Helds J.K., Stothard J.R., Reinhard-Rupp J. (2016). Female genital schistosomiasis (FGS): From case reports to a call for concerted action against this neglected gynaecological disease. Int. J. Parasitol..

[143] Sadeghi-Bojd S., Naghshizadian R., Mazaheri M., Ghane Sharbaf F., Assadi F. (2020). Efficacy of probiotic prophylaxis after the first febrile urinary tract infection in children with normal urinary tracts. J. Pediatr. Infect. Dis. Soc..

[144] Friedrich V., Choi H.W. (2022). The urinary microbiome: Role in bladder cancer and treatment. Diagnostics.

[145] Klein S.L., Flanagan K.L. (2016). Sex differences in immune responses. Nat. Rev. Immunol..

[146] Asare K.K., Afful P., Abotsi G.K., Adu-Gyamfi C.O., Benyem G., Katawa G., Arndts K., Ritter M. (2024). Schistosomiasis Endemicity and its Role in Sexually Transmitted Infections-A Systematic Review and Meta-analysis. Front. Parasitol..

[147] da Paz V.R., Figueiredo-Vanzan D., dos Santos Pyrrho A. (2019). Interaction and involvement of cellular adhesion molecules in the pathogenesis of Schistosomiasis mansoni. Immunol. Lett..

[148] Liu Z., Zhang L., Liang Y., Lu L. (2022). Pathology and molecular mechanisms of *Schistosoma japonicum*-associated liver fibrosis. Front. Cell. Infect. Microbiol..

[149] Partida-Rodríguez O., Serrano-Vázquez A., Nieves-Ramírez M.E., Moran P., Rojas L., Portillo T., González E., Hernández E., Finlay B.B., Ximenez C. (2017). Human intestinal microbiota: Interaction between parasites and the host immune response. Arch. Med. Res..

[150] Stark K.A., Rinaldi G., Cortés A., Costain A., MacDonald A.S., Cantacessi C. (2023). The role of the host gut microbiome in the pathophysiology of schistosomiasis. Parasite Immunol..

[151] Stark K.A., Rinaldi G., Costain A., Clare S., Tolley C., Almeida A., McCarthy C., Harcourt K., Brandt C., Lawley T.D. (2024). Gut microbiota and immune profiling of microbiota-humanised versus wildtype mouse models of hepatointestinal schistosomiasis. Anim. Microbiome.

[152] Martin I., Kaisar M.M., Wiria A.E., Hamid F., Djuardi Y., Sartono E., Rosa B.A., Mitreva M., Supali T., Houwing-Duistermaat J.J. (2019). The effect of gut microbiome composition on human immune responses: An exploration of interference by helminth infections. Front. Genet..

[153] Cortés A., Martin J., Rosa B.A., Stark K.A., Clare S., McCarthy C., Harcourt K., Brandt C., Tolley C., Lawley T.D. (2022). The gut microbial metabolic capacity of microbiome-humanized vs. wild type rodents reveals a likely dual role of intestinal bacteria in hepato-intestinal schistosomiasis. PLoS Negl. Trop. Dis..

[154] Zaghloul M.S., Zaghloul T.M., Bishr M.K., Baumann B.C. (2020). Urinary schistosomiasis and the associated bladder cancer: Update. J. Egypt. Natl. Cancer Inst..

[155] Grondin J.A., Jamal A., Mowna S., Seto T., Khan W.I. (2024). Interaction between Intestinal Parasites and the Gut Microbiota: Implications for the Intestinal Immune Response and Host Defence. Pathogens.

[156] Lacorcia M., Prazeres da Costa C.U. (2018). Maternal Schistosomiasis: Immunomodulatory effects with lasting impact on allergy and vaccine responses. Front. Immunol..

[157] Whiteside S.A., Razvi H., Dave S., Reid G., Burton J.P. (2015). The microbiome of the urinary tract—A role beyond infection. Nat. Rev. Urol..

