# Supplementary material for: Schistosomiasis–Microbiota Interactions: A Systematic Review and Meta-Analysis
Source: Pathogens. 2024 Oct 16;13(10):906. doi: 10.3390/pathogens13100906 (PMC11510367; doi:10.3390/pathogens13100906)
Supplement: Supplementary file 1 [file pathogens-13-00906-s001.zip › pathogens-3222002-supplementary.pdf]

Table S1. PRISMA Checklist

Section/topic	#	Checklist item	Reported on page #
**TITLE**			
Title	1	Identify the report as a systematic review, meta-analysis, or both.	1
**ABSTRACT**			
Structured summary	2	Provide a structured summary including, as applicable: background; objectives; data sources; study eligibility criteria, participants, and interventions; study appraisal and synthesis methods; results; limitations; conclusions and implications of key findings; systematic review registration number.	2
**INTRODUCTION**			
Rationale	3	Describe the rationale for the review in the context of what is already known.	3
Objectives	4	Provide an explicit statement of questions being addressed with reference to participants, interventions, comparisons, outcomes, and study design (PICOS).	3-6
**METHODS**			
Protocol and registration	5	Indicate if a review protocol exists, if and where it can be accessed (e.g., Web address), and, if available, provide registration information including registration number.	-
Eligibility criteria	6	Specify study characteristics (e.g., PICOS, length of follow-up) and report characteristics (e.g., years considered, language, publication status) used as criteria for eligibility, giving rationale.	7
Information sources	7	Describe all information sources (e.g., databases with dates of coverage, contact with study authors to identify additional studies) in the search and date last searched.	5
Search	8	Present full electronic search strategy for at least one database, including any limits used, such that it could be repeated.	6
Study selection	9	State the process for selecting studies (i.e., screening, eligibility, included in systematic review, and, if applicable, included in the meta-analysis).	8
Data collection process	10	Describe method of data extraction from reports (e.g., piloted forms, independently, in duplicate) and any processes for obtaining and confirming data from investigators.	8
Data items	11	List and define all variables for which data were sought (e.g., PICOS, funding sources) and any assumptions and simplifications made.	7-8
Risk of bias in individual studies	12	Describe methods used for assessing risk of bias of individual studies (including specification of whether this was done at the study or outcome level), and how this	8

		information is to be used in any data synthesis.	
Summary measures	13	State the principal summary measures (e.g., risk ratio, difference in means).	9
Synthesis of results	14	Describe the methods of handling data and combining results of studies, if done, including measures of consistency (e.g., I^2) for each meta-analysis.	9

Section/topic	#	Checklist item	Reported on page #
Risk of bias across studies	15	Specify any assessment of risk of bias that may affect the cumulative evidence (e.g., publication bias, selective reporting within studies).	10
Additional analyses	16	Describe methods of additional analyses (e.g., sensitivity or subgroup analyses, meta-regression), if done, indicating which were pre-specified.	9-10
**RESULTS**			
Study selection	17	Give numbers of studies screened, assessed for eligibility, and included in the review, with reasons for exclusions at each stage, ideally with a flow diagram.	11
Study characteristics	18	For each study, present characteristics for which data were extracted (e.g., study size, PICOS, follow-up period) and provide the citations.	11
Risk of bias within studies	19	Present data on risk of bias of each study and, if available, any outcome level assessment (see item 12).	11
Results of individual studies	20	For all outcomes considered (benefits or harms), present, for each study: (a) simple summary data for each intervention group (b) effect estimates and confidence intervals, ideally with a forest plot.	11
Synthesis of results	21	Present results of each meta-analysis done, including confidence intervals and measures of consistency.	11-16
Risk of bias across studies	22	Present results of any assessment of risk of bias across studies (see Item 15).	17
Additional analysis	23	Give results of additional analyses, if done (e.g., sensitivity or subgroup analyses, meta-regression [see Item 16]).	11-17
**DISCUSSION**			
Summary of evidence	24	Summarize the main findings including the strength of evidence for each main outcome; consider their relevance to key groups (e.g., healthcare providers, users, and policy makers).	18-24
Limitations	25	Discuss limitations at study and outcome level (e.g., risk of bias), and at review-level (e.g., incomplete retrieval of identified research, reporting bias).	-
Conclusions	26	Provide a general interpretation of the results in the context of other evidence, and implications for future research.	24
**FUNDING**			

Funding	27	Describe sources of funding for the systematic review and other support (e.g., supply of data); role of funders for the systematic review.	-

Table S2. Literature Search Strategy

No	Database	Search query	Hits	Filters
1	PubMed	(("schistosomiasis"[MeSH Terms] OR "schistosomiasis"[All Fields] OR "schistosomiases"[All Fields]) AND ("bacteria s"[All Fields] OR "bacteriae"[All Fields] OR "bacterias"[All Fields] OR "microbiology"[MeSH Subheading] OR "microbiology"[All Fields] OR "bacteria"[All Fields] OR "bacteria"[MeSH Terms]) AND ("coinfection"[MeSH Terms] OR "coinfection"[All Fields] OR ("co"[All Fields] AND "infection"[All Fields]) OR "co infection"[All Fields])) AND ((ffrft[Filter]) AND (fha[Filter]) AND (fft[Filter]) AND (1965/1/1:2024/6/1[pdat]))<br>Translations schistosomiasis: "schistosomiasis"[MeSH Terms] OR "schistosomiasis"[All Fields] OR "schistosomiases"[All Fields] bacteria: "bacteria's"[All Fields] OR "bacteriae"[All Fields] OR "bacterias"[All Fields] OR "microbiology"[Subheading] OR "microbiology"[All Fields] OR "bacteria"[All Fields] OR "bacteria"[MeSH Terms]<br>co-infection: "coinfection"[MeSH Terms] OR "coinfection"[All Fields] OR ("co"[All Fields] AND "infection"[All Fields]) OR "co infection"[All Fields]	135	Abstract, Free full text, Full text, (1965 to 2024)
2	Scopus	TITLE-ABS-KEY (SCHISTOSOMIASIS AND BACTERIAL AND CO-INFECTIONS) AND PUBYEAR > 1996 AND PUBYEAR < 2024	25	Search from 1996 to 2024
3	MedlinePlus	Schistosomiasis and bacteria infection	4	
4	ClinicalTrials.gov	Condition/disease: Schistosomiasis<br>Other terms: bacterial co-infections	1	Study starts from 01/01/1965 to

				01/01/2024
5	Cochrane	Schistosomiasis and bacterial co-infections	0	
	Total		165	

Table S3. Joanna Briggs Institute critical appraisal checklist guidelines for Quality assessment

Term		Definition
Critical appraisal		The process of systematically assessing the outcome of scientific research to judge its trustworthiness, value, and relevance in a particular context.
External validity		Applicability of the findings to a given population.
Generalizability		The degree to which the results of a study can be applied to a broader population or situation.
Imprecision		The GRADE approach to rating imprecision focuses on the 95% CI around the best estimate of the absolute effect.
Indirectness		The GRADE approach to rating indirectness focuses on concerns about how the population, intervention, or outcomes differ from those of interest.
Internal validity		A measure of how well a study is conducted and how accurately its results reflect the studied group.
Methodological quality		The extent to which there is potential for errors and bias in the design and execution of a study.
Power		The probability of finding a statistically significant result.
Publication bias		The likelihood studies have not been published based on the outcome of the research study.
Random error		An error in measurement caused by factors that vary from one measurement to another.
Reporting		The extent to which a complete and transparent description of the design,

quality		conduct, and analysis of a study is given.
Risk of bias		The likelihood that features of the study design or conduct of the study will give misleading results.
Sample size		The number of participants or observations included in a study.
Statistical conclusion validity		The extent to which the conclusions of research are founded on adequate analysis of the data.
Systematic error		Errors that affect the accuracy of a measurement or cause readings to differ from the true value by a consistent amount each time a measurement is made.

Table S4. Quality assessment results

			Quality criteria score									
**SN**	**Study**	**Country**	**A**	**B**	**C**	**D**	**E**	**F**	**G**	**H**	**I**	**Total**
1	Kone et al. [43]	Nigeria	1	1	1	1	1	1	1	1	1	9
2	Seynabou et al. [44]	Senegal	1	1	1	1	1	1	1	1	1	9
3	Eyong et al. [45]	Nigeria	1	1	1	1	1	1	1	1	1	9
4	Usaini et al. [46]	Nigeria	1	1	1	1	1	1	1	1	1	8
5	Ossai et al. [47]	Nigeria	1	1	1	1	1	1	1	1	1	9
6	Dada et al. [48]	Nigeria	1	1	1	1	1	1	1	1	1	9
7	Hicks et al. [49]	Egypt	1	1	1	0	1	1	1	0	1	7
8	Laughlin et al. [50]	Egypt	1	1	1	0	1	1	1	1	1	8
9	F. X. Pi-Sunyer et al. [51]	Nigeria	1	1	1	1	1	1	1	1	1	9

10	Du et al. [52]	China	1	1	1	1	1	1	1	1	1	9
11	Mbuyi-Kalonji et al. [53]	Democratic Republic of Congo	1	1	1	1	1	1	1	1	1	9
12	Marege et al. [54]	Ethiopia	1	1	1	1	1	1	1	1	1	9
13	Bishop et al. [55]	Nigeria	1	1	1	1	1	1	1	1	1	9
14	Oyedeji et al. [56]	Nigeria	1	1	1	0	1	1	1	1	1	8
15	Gendrel et al. [57]	Gabon	1	1	1	0	1	1	1	1	1	8
16	Gadoth et al. [58]	Democratic Republic of Congo	1	1	1	1	1	1	1	1	1	9
17	Mhimbira et al. [59]	Tanzania	1	1	1	1	1	1	1	1	1	9
18	Mduluza-Jokonya et al. [60]	Zimbabwe	1	1	1	1	1	1	1	1	1	9
19	Bullington et al. [61]	Tanzania	1	1	1	0	1	1	1	1	1	8
20	A Ologunde et al. [62]	Nigeria	1	1	1	1	1	1	1	1	1	9
21	Mohamed et al. [63]	Sudan	1	1	1	1	1	1	1	1	1	9
22	Mduluza-Jokonya et al. [64]	Zimbabwe	1	1	1	1	1	1	1	1	1	9
23	Range et al. [65]	Tanzania	1	1	1	1	1	1	1	1	1	9
24	Abate et al. [66]	Ethiopia	1	1	1	1	1	1	1	1	1	9
25	Mohager et al. [67]	Saudi Arabia	1	1	1	0	1	1	1	1	1	8
26	Dennison et al. [68]	Brazil	1	1	1	1	1	1	1	1	1	9
27	Owino [69]	Kenya	1	1	1	1	1	1	1	1	1	9
28	Amoo et al. [70]	Nigeria	1	1	1	1	1	1	1	1	1	9
29	Yohanna et al. [71]	Nigeria	1	1	1	1	1	1	1	1	1	9

30	Yirenya-Tawiah et al. [72]	Ghana	1	1	1	1	1	1	1	1	1	9
31	Aniekwe et al. [73]	Nigeria	1	1	1	1	1	1	1	1	1	8
